# Supplementary material for: Views of prison staff in Scotland on the potential benefits and risks of e-cigarettes in smoke-free prisons: a qualitative focus group study
Source: BMJ Open. 2019 Jun 25;9(6):e027799. doi: 10.1136/bmjopen-2018-027799 (PMC6597633; doi:10.1136/bmjopen-2018-027799)
Supplement: Author's manuscript [file bmjopen-2018-027799.draft_revisions.pdf]

BMJ Open is committed to open peer review. As part of this commitment we make the peer review history of every article we publish publicly available.

When an article is published we post the peer reviewers' comments and the authors' responses online. We also post the versions of the paper that were used during peer review. These are the versions that the peer review comments apply to.

The versions of the paper that follow are the versions that were submitted during the peer review process. They are not the versions of record or the final published versions. They should not be cited or distributed as the published version of this manuscript.

BMJ Open is an open access journal and the full, final, typeset and author-corrected version of record of the manuscript is available on our site with no access controls, subscription charges or pay-per-view fees (http://bmjopen.bmj.com).

If you have any questions on BMJ Open's open peer review process please email info.bmjopen@bmj.com

BMJ Open

# Views of prison staff in Scotland on the potential benefits and risks of e-cigarettes in smoke-free prisons

| | |
|---|---|
| Journal: | *BMJ Open* |
| Manuscript ID | bmjopen-2018-027799 |
| Article Type: | Research |
| Date Submitted by the Author: | 14-Nov-2018 |
| Complete List of Authors: | Brown, Ashley; University of Stirling Institute for Social Marketing,<br>Sweeting , Helen ; University of Glasgow, MRC/CSO Social & Public Health Sciences Unit<br>Semple, Sean; University of Stirling, Institute of Social Marketing<br>Bauld, Linda; University of Stirling, Stirling Management School<br>Demou, Evangelia; University of Glasgow, MRC/CSO Social & Public Health Sciences Unit<br>Logan, Greig; University of Glasgow, 4. Institute of Health and Wellbeing<br>Hunt, Kate; University of Stirling, Institute for Social Marketing |
| Keywords: | PUBLIC HEALTH, Health policy < HEALTH SERVICES ADMINISTRATION & MANAGEMENT, QUALITATIVE RESEARCH |
| | |

SCHOLARONE™
Manuscripts

**Views of prison staff in Scotland on the potential benefits and risks of e-cigarettes in smoke-free prisons**

Ashley Brown, MA1,2

Helen Sweeting, PhD2

Sean Semple, PhD1

Linda Bauld, PhD,1,3

Evangelia Demou, PhD2

Greig Logan,4

Kate Hunt, PhD1,2

Corresponding author: Ashley Brown, a.l.brown@stir.ac.uk

(Text currently ~4235 without title page and abstract)

1. Institute for Social Marketing, Faculty of Health Sciences and Sport, University of Stirling, Pathfoot Building, Stirling, FK9 4LA.
2. MRC/CSO Social and Public Health Sciences Unit, 200 Renfield St, University of Glasgow, G2 3AX
3. UK Centre for Tobacco and Alcohol Studies, University of Stirling, UK
4. Institute of Health and Wellbeing, University of Glasgow, Glasgow G12 8RZ.

For peer review only

# ABSTRACT

## Objective

Electronic cigarettes (e-cigarettes) were first introduced into all Scottish prisons in February 2018, some months after the prison system began preparing for a smoking ban in November 2018. This paper aimed to explore prison staff views on the potential benefits and risks of e-cigarettes accompanying the implementation of smoke-free prison polices.

## Setting

Fourteen prisons in Scotland.

## Participants

Seventeen focus groups and two paired interviews were conducted with 132 staff in 14 Scottish prisons 4-9 months before a smoking ban was announced in July 2017. Both smoking and non-smoking staff were invited to participate.

## Results

Prison staff highlighted three potential risks of e-cigarettes in a smoke-free prison: staff health risks from exposure to second-hand vapour; prisoner health risks from vaping; and risks to both groups from device misuse, product defects or accidents. Conversely, potential benefits of e-cigarettes in smoke-free prisons centred on: reducing smoking-related health harms to staff and prisoners; helping prisoners to quit tobacco; and supporting staff to maintain safety and discipline in prison. Staff opinions about e-cigarettes were formed against a background of lack of experience of vaping and some uncertainty and misunderstandings about e-cigarettes.


For peer review only

## Conclusion

Our findings reflect the public health challenges of communicating the benefits and risks surrounding e-cigarettes against a backdrop of gaps in evidence and some uncertainty and misunderstanding in the public mind about vaping. The complexity of prisons as workplaces and the distinctive nature of prison tobacco control policy and culture may create further challenges in communication with prison staff on e-cigarettes in prisons.

## STRENGTHS AND LIMITATIONS OF THIS STUDY

- To our knowledge, this is the first study to examine staff views on the potential benefits and risks of introducing e-cigarettes for prisoners in a smoke-free prison system, using data collected prior to the announcement of a smoking ban and before there was widespread experience of e-cigarette use in prison systems.
- This study provides valuable insight into the potential place of e-cigarettes in a complex workplace setting in which staff were, on the one hand, still exposed to second-hand smoke, whilst on the other increasingly aware that they could be responsible for the management and care of vulnerable and/or challenging individuals subject to enforced smoking abstinence.
- Our results are based on analysis of rich qualitative data collected from a relatively large sample of prison staff from fourteen Scottish prisons with varied smoking histories and experience of working with diverse prisoner groups.
- It is important to acknowledge that the sample were self-selecting, leading to an under-representation of staff with direct experience of vaping, and that perspectives may have developed since these qualitative data were collected.

For peer review only

# INTRODUCTION

There is a growing consensus that using e-cigarettes (vaping) is safer than smoking conventional cigarettes. A review of the scientific evidence on e-cigarettes, conducted by the National Academies of Sciences, Engineering and Medicine,[1] reported "*conclusive evidence that completely substituting e-cigarettes for combustible tobacco cigarettes reduces users' exposures to numerous toxins and carcinogens present in combustible tobacco cigarettes*." A similar view on e-cigarettes has been reached by UK health organisations, including, for example, the Royal College of Physicians,[2] Public Health England[3 4] and NHS Health Scotland.[5] There is however, greater disagreement within academic and health communities over issues such the effectiveness of e-cigarettes as a smoking cessation aid[6] and the potential influence of e-cigarettes on smoking norms and uptake among adults and young people.[1 7]

The contested nature of e-cigarettes is perhaps unsurprising. The introduction of a new technology is inevitably accompanied by a period of scientific uncertainty and debate about how to regulate technology in the absence of conclusive evidence about its health effects. The 'precautionary principle' may be relevant in situations of uncertainty. The WHO's definition of the principle centres on the idea that "*…scientific uncertainty should not be used as a reason to postpone preventive measures*".[8] The precautionary principle requires complex judgements (even conjectures) about the likely balance of potential benefits and risks of implementation of different types of preventive measures vs. not implementing these measures.[9] With respect to e-cigarettes, Fairchild and Bayer[10] suggest that beliefs among some experts that e-cigarettes are a potential threat to health and require strict regulation are *"shaped implicitly by a precautionary impulse"*. On the other hand, it has been suggested that experts who believe that e-cigarettes are part of the solution to the challenge of tobacco control might be influenced by the principle of harm reduction.[10] Harm reduction strategies

For peer review only

are informed by a belief that steps should be taken to minimise harm from tobacco and other drug use in circumstances in which abstinence is not achievable.[11]

In the UK, it is illegal to smoke in most enclosed workplaces and public spaces. As both residential settings and workplace environments, prisons have historically adopted a distinctive approach to tobacco control, including partial exemption from smoke-free laws in the UK.[12] However, ongoing concerns about SHS exposures are one factor in recent decisions by the UK and Scottish governments to extend smoke-free policies to all areas of prisons in Wales and England (rolling out from 2016) and Scotland (from 30th November 2018).

Contrasting with the legal restrictions on smoking in public places, employers and organisations are permitted to develop policies on vaping which suit their specific circumstances. E-cigarettes have been available for prisoners to buy in some English and Welsh prisons from 2016 and were first made available for prisoners to buy in all Scottish prisons in early 2018 (after the data presented in this paper were collected). The potential for e-cigarettes to help some prisoners to remain smoke-free is recognised by organisations such as NHS Health Scotland,[13] while other commentators have discussed the potential negative health and organisational effects of selling e-cigarettes in prisons which have implemented smoking bans.[14 15]

Commentary and research on e-cigarettes and their place in smoke-free environments has largely focused on the opinions of public health experts and the general public.[16 17] Specific research examining employees' views on vaping in particular settings/workplaces is lacking. We believe the Tobacco In Prisons study (TIPs), as reported here, is one of the first studies to address specific evidence gaps in respect of e-cigarettes in one workplace, prisons, and to investigate views on the potential role of e-cigarettes in accompanying the removal of tobacco across a country's prison system.  In this qualitative paper we present prison staff

For peer review only - http://bmjopen.bmj.com/site/about/guidelines.xhtml

views, using data from Phase 1 of TIPs. These data were collected from 132 prison staff in 14 Scottish prisons several months prior to the July 2017 announcement that a comprehensive ban on smoking would be introduced from 30th November 2018 and prior to any significant policy debate in Scotland about the sale of e-cigarettes to prisoners. Other TIPs Phase 1 data, on prison staff and prisoner views on prison smoking bans[18] and levels of SHS in all Scottish prisons,[19] are reported elsewhere.

# METHODS

Data were collected prior to the announcement of the intention to implement a smoking ban in Scottish prisons. At the time of data collection, prisoners were allowed to smoke tobacco in cells and outdoor areas; staff could not smoke anywhere on prison premises. Nobody was permitted to vape in Scottish prisons during the period in which these data were collected (November 2016-April 2017).

The design, methods and tools for TIPs were extensively discussed with a specially convened Scottish Prison Service (SPS) Research Advisory Group, and approved by the SPS Research Access and Ethics Committee and University of Glasgow's College of Social Sciences Ethics Committee (ref number relating to staff data: 400150214).

## Patient and Public Involvement

The TIPs study was designed to ensure that the views of prisoners and prison staff, as expressed to a research team who were independent of the prison service, could be heard at different stages of the process of moving towards smoke-free prisons in Scotland. At all stages of the study, a Research Advisory Group which included staff from various parts of the prison service and representation from unions representing prison staff, has given extensive

For peer review only

feedback on the overall design of the study and on study materials (including topic guides for qualitative interviews/focus groups, and survey questionnaires).

## Sampling and recruitment

As reported elsewhere,[18] seventeen focus groups and two paired interviews (hereafter referred to collectively as ‘focus groups’) were conducted with staff from 14 Scottish prisons who had been recruited through a point of contact in each prison. We asked the point of contact to invite both smoking and non-smoking staff to participate. Of 132 participating staff, 78 had never smoked (NS); 31 were ex-smokers (ExS), and 10 currently used tobacco cigarettes. The smoking status for 13 participants is not known (NK). Seven staff reported having ever used an e-cigarette: four were currently vaping (V) and three were no longer vaping (ExV). Quotations are included to illustrate key perspectives, indicating the prison code, focus group and smoking status of each speaker (e.g. KA04 S=prison K, group A, participant 04, Smoker). Codes were randomly allocated to prisons by the research team for this paper to protect anonymity.

## Data collection

Focus groups (range 5-12 (mode 6) participants) were conducted between November 2016 and April 2017 by a member of the TIPs research team. The topic guide covered: smoking and exposures to SHS, particularly within prisons; smoking norms and perceived prevalence within the prison; the ‘culture’ of smoking within prisons; management of nicotine addiction (including e-cigarettes) in prisons and wider society; and opinions on restrictions on smoking.[18] Specific areas for discussion on e-cigarettes included: whether staff had used e-cigarettes or knew others who used e-cigarettes; opinions about e-cigarettes in general; views on what might be good or bad about prisoners or staff vaping in prison; and opinions on any issues which might be raised by allowing vaping in prisons. The researchers used the guide,

For peer review only

formulating questions using their own words (often in response to issues raised by the groups), adjusting the order of topics as appropriate; they prompted further discussion where relevant and invited staff to raise any points which they thought were pertinent.

Phase 1 survey data were also collected online from 1,271 (estimated 27% return) staff in November-December 2016, prior to the announcement of the forthcoming prison smoking ban. The questionnaire included sections on similar (but not identical) topics to the staff focus groups; full details of the methodology of the online survey are reported elsewhere.[18]

### Data analysis

With written consent from participants, focus groups were audio recorded and transcribed verbatim. Transcripts were checked and de-identified prior to management of the data. TIPs researchers (KH, HS, ED and GL) who conducted the fieldwork developed a descriptive coding scheme which included e-cigarettes; other themes are reported elsewhere[18]. Detailed summaries were created by AB for all data coded to the 'e-cigarettes' theme using the Framework function in Nvivo software (QSR international). Data summaries and excerpts were thematically analysed and a summary of the key themes in these data was produced by AB. All authors agreed on the final themes. To place these qualitative data in context, we first briefly report relevant results from the TIPs Phase 1 staff survey, which included questions on prison smoking bans and e-cigarettes.

## RESULTS

As reported elsewhere,[18] the online staff survey showed that 74% of staff (strongly) agreed that 'prison smoking bans are a good idea'. The proportion who (strongly) agreed that 'prison smoking bans are ok if prisoners are allowed e-cigarettes or vapes' was 36%. In addition,

For peer review only

staff were asked whether e-cigarettes should be: 'available for prisoners to buy on the canteen?'; 'provided on a temporary basis to help with tobacco withdrawal when prisoners are admitted to prison?'; and 'available to prisoners to help them to stop smoking?' Yes vs no responses to these three questions were split almost 50/50 (yes responses 53%, 47% and 54% respectively), indicating that staff were divided on their views whether e-cigarettes should be available to prisoners at that stage. Their more detailed views, as expressed in the focus groups, are presented below.

## Personal experience and expressed knowledge of e-cigarettes: staff focus groups

Most staff who participated in the focus groups had little personal experience of e-cigarettes; only a small number (n=7/132) reported having ever tried vaping. Consequently, a notable feature across the group discussions was expressed low levels of knowledge about e-cigarettes, which appeared to be a source of uncertainty and some confusion. One way in which this emerged was in the different names which staff gave to e-cigarettes, including "*vaping machines*" (JA05 NS), "*vapour sticks*" (FA06 NS), and "*vape cigarette*" (HA03 ExS). Another way in which uncertainty about e-cigarettes emerged was in the frequent questions which staff asked each other or the interviewer during discussions:

HA05 NS *Isn't it quite expensive as well e-cigarettes, are they not quite expensive?*
HA02 NS *I don't know, I think they're getting cheaper, they were quite expensive.*

Specifically, there were instances where staff were unsure about how e-cigarettes work and whether there is more than one type of product. Staff occasionally muddled 'tobacco' and 'nicotine' when discussing e-cigarettes, although mistakes in terminology were generally

For peer review only

corrected by another member of the group. There were also examples where staff appeared to confuse e-cigarettes with a nicotine inhaler.

Uncertainty was also expressed by some staff about what is known about the health risks and safety of e-cigarettes:

FA01 NS "*[w]ell I was just gonna ask, why are they banning vaping [in other contexts outside of prisons], is there something wrong with vaping? Because they're banning it in lots of different places…for some reason*"

In other cases, staff expressed greater awareness that there are gaps in the evidence on e-cigarettes, which was another source of uncertainty. It is interesting to note however, that some staff seemed unfamiliar with the pace at which knowledge has accumulated in recent years:

BA10 NS *The last time they reviewed the policy, there was still no reliable research on the health implications of e-cigarettes.*

Apparent misunderstandings about e-cigarettes were also identified. These included a staff member believing that it was more dangerous for non-smokers to take up e-cigarettes compared to conventional smoking and statements made as 'facts', such as that e-cigarettes "*emit ten carcinogens as opposed to 100 from ordinary cigarettes* (CD27 ExS).

Hence, it was against this background that staff were evaluating the potential benefits and risks of vaping in prison if tobacco were to be removed. Overall opinions around allowing e-cigarettes in a smoke-free prison included views which could be described as positive and highly negative, with some staff reporting that they did not feel sufficiently well informed to have an opinion. Their perceptions of the key potential risks and benefits of e-cigarettes in a smoke-free prison are explored in detail below; this extends an earlier paper reporting on staff

For peer review only

views on prison smoking bans more generally, which included only brief mention of e-cigarettes.[18]

## Perceptions of potential risks of e-cigarettes in a smoke-free prison

Potential risks of e-cigarettes in a smoke-free prison centred on three themes, described below.

### Potential risks to staff health from second-hand vapour

Staff worried that they might be harmed by breathing the vapour from prisoners' e-cigarettes when they came to work. Several statements were made that health risks from second-hand vapour exposures might be uncovered in the future:

| | |
|---|---|
| *LA8 ExS* | *…what's in that [e-cigarette vapour]? You obviously don't know, so I think in years to come they'll [bystanders] end up having problems. Might not be as bad as cigarette smoke, but I think the time to come…when they start doing research like that.* |

Consequently, there were some staff who expressed beliefs that e-cigarettes should be prohibited in Scottish prisons as a protective measure. Precautionary thinking about second-hand vapour (and the use of e-cigarettes) appeared to be influenced by several factors. As discussed, some staff appeared aware that there are gaps in current scientific understanding of the health effects of e-cigarettes and second-hand vapour.

| | |
|---|---|
| *CC19 NS* | *I don't think there's enough research been done on the e-cigarettes to see if you can get passive smoking through that either so I wouldn't be happy with smokers …* |
| *CC22 NS* | *So ban it until you've proved it's safe.* |
| *Interviewer* | *So they're basically too new at the moment?* |
| *CC22 NS* | *Yes, that's it. You don't allow it until you can prove it's safe. This is what has not happened with tobacco.* |

For peer review only

The restrictions that are in place on vaping in many public places appeared to reinforce perceptions that second-hand vapour might pose a danger to health:

*NA06, S/ExV* *It's the vapour itself. Which is why obviously it's been banned on trains and things like that, because they don't know enough about the vapour and the affect it might have on those around them. So you might be trading second hand smoke for second hand vapour. And it's…could be having exactly the same effect on people's lungs and everything else as the second hand smoke does, so…*

A desire to avoid past mistakes which had been made in tobacco control, perceptions that "*we don't know exactly what's in e-cigarettes*" (KA02 NS) and beliefs that prisoners might use poor quality products are examples of further justifications given by staff for continuing to disallow vaping in prisons.

## Potential risks to prisoner health from vaping

Some staff worried about potential risks to prisoner health from the use of e-cigarettes, given that the long-term effects of vaping on the body are not yet fully known. For example, one member of staff stated "*I just don't think we should be giving them [prisoners] something [e-cigarettes] as a substitute that we don't even know a hundred per cent about.*" (AA06 NS). Two other group members continued this discussion, saying:

*AA07 ExS* *Ten years down the line, we could all be smoking these vapes, and then we find out there's a risk.*

*AA01 NS* *A brain tumour, or something.*

Concerns about risks to prisoner health from vaping might also have been influenced by perceptions that e-cigarettes were being used, in wider society, as a long-term replacement for tobacco, rather than as a means to cut down and then quit nicotine. There were some suggestions that prisoners might struggle to give up vaping and that 'addiction' to e-cigarettes

For peer review only

was undesirable on health grounds. There was a suggestion that it might be beneficial if prisoners had access to e-cigarettes on an interim (rather than permanent) basis to help them to manage cravings without forming a vaping habit. However, there were other staff who suggested that this might be unfair/ineffectual for prisoners who do not want to become abstinent.

Potential risks to staff and prisoners from device misuse, product defects or accidents

As misuse of permitted items was perceived to be an integral part of prison culture, there was a significant amount of discussion about potential ways in which prisoners might attempt to find alternative uses for e-cigarettes.

NA06 S/ExV *Nothing's a hundred per cent tamper-proof and we're never going to find anything that's a hundred per cent tamper-proof…*

Specific concerns focussed on rechargeable devices and associated chargers and risk of devices being used to conceal contraband, 'smoke' illegal drugs (such as new psychoactive substances), charge prohibited mobile phones, make weapons etc.

EB12 NS *I mean, it's an alternative. And obviously, for your second hand smoke, it's beneficial. But what could they do with these cartridges, and that sort of stuff? I mean, you're talking about people who can make anything out of anything…Is this just more stuff you're introducing to the jail, which they could use to do whatever?*

There was also discussion about potential risk of injury to staff and prisoners if devices were to leak, explode or catch fire. For example, one member of staff stated that e-cigarettes had received 'bad press' for '*blowing up in people's faces and people maybe have them in their pocket and it leaks or something and it burns"* (CB01 NS).

For peer review only

## Perceptions of potential benefits of e-cigarettes in a smoke-free prison

While risks were identified, so were potential benefits of allowing e-cigarettes in a smoke-free prison, described below.

### Reducing harm to staff and prisoner health from smoking and second-hand smoke

E-cigarettes were perceived by some staff to contain fewer harmful chemicals compared with conventional cigarettes and thus to be likely to pose fewer risks to the health of users:

| | |
|---|---|
| JA02 NS | *I think they [e-cigarettes] are better for…smokers that want to come off smoking and they change from cigarettes to them because there are less carcinogens in but there is still something in them [e-cigarettes]. It has to be to create the vapour to carry the nicotine there's something in there.* |

Some staff also believed that second-hand vapour might be less dangerous and unpleasant for bystanders, with one staff member even saying the smell produced by some e-liquids was "*quite nice*" (FA02, NK). Consequently, there was a suggestion that e-cigarettes might be beneficial for the health of everyone working and living in prisons when weighed against the dangers of smoking and second-hand smoke:

| | |
|---|---|
| *FA02 unknown* | *They could get the vapour thing, I don't know a lot about it, but vaping is safer than smoking. Why not let them vapour when they're in prison.* |
| *FA05 NS* | *I'm not being funny...* |
| *FA02 unknown* | *Better for us as well.* |
| *FA05 NS* | *...I would rather somebody was vaping in the jail, than smoking some of the crap they smoke.* |
| *FA02 unknown* | *Yeah.* |
| *FA03 NS* | *And it's got to be better for the smoker* |

Some expressed the view that, as a matter of fairness, staff should be allowed to use e-cigarettes in designated areas at work if prisoners were permitted to vape in the future.

## Helping prisoners to quit tobacco

Some suggested that e-cigarettes could play a role in helping prisoners to manage without tobacco should smoking be banned in prison, including individuals who intended to return to smoking after their smoke-free prison sentence. There was some discussion about the extent to which allowing prisoners to vape in prison was consistent with current practice in respect of treatment of prisoners addicted to other substances, such as illegal drugs like heroin.

IA02 V *"I totally agree [about the introduction of e-cigarettes into prisons]. I think it's been borne out with other…with the way we've treated other addictions, mainly methadone, I think there does need to be a substitute, it's by all accounts…the research thus far says it's a far, far cleaner substitute…*

Lack of availability of 'medical' e-cigarettes in the UK, and uncertainties about the health effects of vaping were highlighted as points of difference between e-cigarettes and other 'substitute' products such as methadone. Additionally, some implied that existing nicotine replacement products could fulfil a similar role to e-cigarettes (this theme is explored more broadly elsewhere[18]).

## The potential role of e-cigarettes in staff strategies to maintain safety and discipline in prison

There were some perceptions that, in the absence of tobacco, nicotine substitutes such as e-cigarettes and nicotine replacement therapy might help staff with the management and care of prisoners who were unable to smoke, especially new arrivals into custody:

GA04 NS *"I think if I was offering some…prisoners, you know, there's your cigarette, I'm taking them off you, there's a lollypop or there's an e-cigarette I think I'd rather give them an e-cigarette"*

Nicotine substitutes, alongside other measures, were also believed to have the potential to reduce potential organisational problems (e.g. incidents of indiscipline and threats to staff safety and operational stability) associated with a ban on tobacco in prisons. However, there was some uncertainty about the degree to which nicotine substitutes might diffuse challenging situations in prisons should tobacco to be removed.

KA02 NS *Well, I think if it's [a smoking ban] managed properly and an alternative [to tobacco] is offered, you know, whether it's a certain e-cigarette or patches or something, then yes, it [a smoking ban] could work, but I think to have an outright ban with no alternative in place would just cause a hell of a problem.*

## DISCUSSION

To our knowledge this is the first study in any country to investigate staff views across an entire prison system on the potential benefits and risks of introducing e-cigarettes for prisoners in a smoke-free prison system. We found evidence of discord among prison staff in Scotland about the overall balance of potential benefits and risks of e-cigarettes in a smoke-free prison setting; it is important to bear in mind that the data were collected well in advance of the announcement of smoke-free prisons in Scotland, and any form of e-cigarettes were available for purchase. We found that concerns about as yet unknown potential risks to health from e-cigarettes and second-hand vapour led some staff to feel apprehensive about the prospect of prisoners' vaping. It is understandable that some prison staff showed precautionary attitudes about the possibility of replacing one workplace hazard (second-hand smoke exposures) with another that they thought could potentially be hazardous (prisoner vaping). The likelihood that some prisoners will try to modify devices, thus possibly causing harm to themselves or others, was cited as another potential risk of e-cigarettes in prisons. Significant discussion among staff about potential device misuse or accidents might be, at

For peer review only

least partly, explained by prison officers' primary responsibilities for maintaining operational safety and stability and concerns about use of new psychoactive substances in UK prisons.[20]

By contrast, staff support for allowing e-cigarettes in a smoke-free prison could be interpreted with reference to the principle of harm reduction. While e-cigarettes were believed to carry some risk, these risks were perceived by some staff to be smaller than the certain dangers of smoking and second-hand smoke and the potential adverse consequences of removing tobacco from prisons. Given ongoing challenges in respect of supporting individuals to abstain from drug and alcohol use in prison, it is understandable that some staff believed that a range of nicotine substitutes should be offered in a smoke-free prison to help in the management of smoking addiction.

Consistent with other qualitative studies,[21-23] our findings suggest some gaps in knowledge, uncertainties, and apparent misunderstandings in the public mind about e-cigarettes. This finding continues to be noteworthy, since there is evidence to suggest that risk perceptions of vaping and second-hand vapour may influence vaping behaviours[24] and shape attitudes towards regulation on the use of e-cigarettes.[16 25] As other commentators have suggested, there continues to be a need for clear and reliable information about e-cigarettes for the public and relevant professionals.[21] We suggest it would be beneficial if specific information were to be developed for prison settings which strikes a balance between taking a clear position on the relative harms of e-cigarettes compared to smoking tobacco or abstinence and acknowledging limitations in the evidence.

A key strength, and novel element, of this paper is that it is based on analysis of rich qualitative data collected from a relatively large sample of prison staff with diverse smoking histories and experiences of working in varied prison settings and with different groups of prisoners. We therefore believe that our results provide a good indication of staff perspectives

For peer review only

on the key potential benefits and risks of e-cigarettes in a smoke-free prison. Our findings may be relevant to prisons in other countries who have adopted, or are considering, similar approaches to the UK on the regulation of tobacco and e-cigarettes.

Our study has three key limitations. First, focus group participants were self-selecting and may not be fully representative of all Scottish prison staff. It is notable that few participating staff (N=7/132) reported current/former vaping experience. A lower rate of current use (~3%) with e-cigarettes among staff focus group participants compared with the general population in Scotland (7%)[26] is perhaps not surprising given that prison staff are not allowed to use e-cigarettes in their place of employment. Second, in some focus groups there were individuals who expressed strong views about whether to allow e-cigarettes in prisons. While TIPs researchers tried to ensure that diverse and opposing positions were captured during the processes of collecting and analysing data and encouraged all participants to speak, it is possible that some people may not have wanted to express their views in front of colleagues and so some positions may not be fully reflected in our findings due to group dynamics. Finally, it is important to acknowledge that staffs' viewpoints on e-cigarettes may have developed since these qualitative data were collected.

Our findings reflect the public health challenges of communicating the benefits and risks surrounding e-cigarettes against a backdrop of gaps in evidence and some uncertainty and misunderstanding in the public mind about vaping. The complexity of prisons as workplaces and the distinctive nature of prison tobacco control policy and culture may create further challenges in communication with prison staff on e-cigarettes in the prison setting.

## CONTRIBUTIONSHIP STATEMENT

KH, HS, SS, ED and LB developed the study. KH and HS conducted most of the focus groups, with input from ED and GL. AB and KH conducted independent initial analyses of

For peer review only

the data, and AB conducted the more detailed framework analysis. AB drafted the manuscript. All authors contributed to interpretation of the data and reviewing of the final paper.

## ACKNOWLEDGEMENTS

We are grateful to the staff who completed the surveys, staff who took part in the focus group discussion, and the Governors in Charge and staff at the Scottish Prison Service and the two non-SPS-run prisons (HMP Addiewell and HMP Kilmarnock) who assisted with the study and facilitated access. We would also like to thank colleagues at the MRC/CSO Public Health Research Facility for help with data management and input (in particular David Walker, Kate Campbell, and Matthew Tolan). We gratefully acknowledge the contribution of our co-investigators in TIPs research team to the overall design of the study (Dr Kathleen Boyd, Dr Philip Conaglen, Dr Peter Craig, Douglas Eadie, Professor Alastair Leyland, Professor Jill Pell,). We would particularly like to thank Sarah Corbett, Linda Dorward, Ruth Parker and members of the SPS Research Advisory Group for their helpful input during the design and conduct of this research.

## COMPETING INTERESTS

None to declare

## FUNDING

The TIPs study was funded by the National Institute for Health Research Public Health Research Programme (project number 15/55/44). HS and ED acknowledge funding from the UK Medical Research Council (MC_UU_12017/12 and MC_PC_13027 to ED) and the Chief Scientist Office (SPHSU-12). The views and opinions expressed are those of the authors and

For peer review only

do not necessarily reflect those of the Public Health Programme, NIHR, NHS or the Department of Health.

## DATA SHARING STATEMENT.

No additional data are available.

For peer review only

# REFERENCES


1. National Academies of Sciences, Engineering and Medicine. Public Health Consequences of E-cigarettes: Conclusions by Outcome https://www.nap.edu/resource/24952/012318ecigaretteConclusionsbyOutcome.pdf (accessed August 2018), 2018.
2. Royal College of Physicians. Nicotine without Smoke: Tobacco harm reduction. https://www.rcplondon.ac.uk/projects/outputs/nicotine-without-smoke-tobacco-harm-reduction-0 (accessed June 2018), 2016.
3. Public Health England. E-cigarettes: a developing public health consensus Accessed online: https://www.gov.uk/government/publications/e-cigarettes-a-developing-public-health-consensus, 2016.
4. McNeill A, Brose LS, Calder R, et al. Evidence review of e-cigarettes and heated tobacco products 2018: A report commissioned by Public Health England. https://assets.publishing.service.gov.uk/government/uploads/system/uploads/attachment_data/file/684963/Evidence_review_of_e-cigarettes_and_heated_tobacco_products_2018.pdf (accessed September 2018): Public Health England 2018.
5. NHS Health Scotland. Consesus statement on e-cigarettes http://www.healthscotland.scot/media/1576/e-cigarettes-consensus-statement_sep-2017.pdf (accessed June 2018), 2017.
6. Villanti AC, Feirman SP, Niaura RS, et al. How do we determine the impact of e-cigarettes on cigarette smoking cessation or reduction? Review and recommendations for answering the research question with scientific rigor. *Addiction* 2018;113(3):391-404. doi: doi:10.1111/add.14020
7. Bauld L, MacKintosh A, Eastwood B, et al. Young People's Use of E-Cigarettes across the United Kingdom: Findings from Five Surveys 2015–2017. *Int J Environ Res Public Health* 2017;14(9):973.
8. Martuzzi M, Tickner JA. The precautionary principle: protecting public health, the environment and the future of our children http://www.euro.who.int/en/publications/abstracts/precautionary-principle-the-protecting-public-health,-the-environment-and-the-future-of-our-children (accessed June 2018): World Health Organisation 2004.
9. Public Health England. Use of e-cigarettes in public places and workplaces: Advice to inform evidence-based policy making. https://assets.publishing.service.gov.uk/government/uploads/system/uploads/attachment_data/file/534586/PHE-advice-on-use-of-e-cigarettes-in-public-places-and-workplaces.PDF (accessed June 2018), 2016.
10. Fairchild AL, Bayer R. Public health. Smoke and fire over e-cigarettes. *Science* 2015;347(6220):375-6. doi: 10.1126/science.1260761 [published Online First: 2015/01/24]
11. Lenton S, Single E. The definition of harm reduction. *Drug Alcohol Rev* 1998;17(2):213-20. doi: doi:10.1080/09595239800187011
12. Scottish Prison Service. Scotland's journey towards smoke-free prisons. http://www.sps.gov.uk/Corporate/Publications/Publication-4405.aspx (accessed September 2018), 2016.
13. NHS Health Scotland. Untitled https://www.scotphn.net/wp-content/uploads/2017/08/2016_10_06-Smokefree-prisons-and-e-cigs-Oct-2016.pdf (accessed June 2018), 2016.
14. Curry L, Lee YO, Rogers T. E-cigarettes made especially for inmates. *Tob Control* 2014;23(e2):e87-e88. doi: 10.1136/tobaccocontrol-2013-051535


For peer review only

15. Young-Wolff KC, Karan LD, Prochaska JJ. Electronic cigarettes in jails: A panacea or public health problem? *JAMA Psychiatry* 2015;72(2):103-04. doi: 10.1001/jamapsychiatry.2014.2224
16. Brose LS, McNeill A, Arnott D, et al. Restrictions on the use of e-cigarettes in public and private places-current practice and support among adults in Great Britain. *Eur J Public Health* 2017;27(4):729-36. doi: 10.1093/eurpub/ckw268
17. Majeed BA, Dube SR, Sterling K, et al. Opinions About Electronic Cigarette Use in Smoke-Free Areas Among U.S. Adults, 2012. *Nicotine Tob Res* 2015;17(6):675-81. doi: 10.1093/ntr/ntu235
18. Brown A, Sweeting H, Logan G, et al. Prison staff and prisoner views on a prison smoking ban: evidence from the Tobacco in Prisons Study. *Nicotine Tob Res* 2018 doi: 10.1093/ntr/nty092 [published Online First: 2018/05/17]
19. Semple S, Sweeting H, Demou E, et al. Characterising the Exposure of Prison Staff to Second-Hand Tobacco Smoke. *Ann Work Expo Health* 2017;61(7):809-21. doi: 10.1093/annweh/wxx058
20. HM Chief Inspector of Prisons for England and Wales. Annual Report 2016–17. https://www.justiceinspectorates.gov.uk/hmiprisons/wp-content/uploads/sites/4/2017/07/HMIP-AR_2016-17_CONTENT_11-07-17-WEB.pdf (accessed August 2018), 2017.
21. Rooke C, Cunningham-Burley S, Amos A. Smokers' and ex-smokers' understanding of electronic cigarettes: a qualitative study. *Tob Control* 2016;25(e1):e60-6. doi: 10.1136/tobaccocontrol-2014-052151 [published Online First: 2015/06/10]
22. Vasconcelos V, Gilbert H. Smokers’ knowledge and perception of electronic cigarettes (e-cigarettes): a qualitative study of non-quitting smokers in a North London general practice. *Prim Health Care Res Dev* 2018:1-9. doi: 10.1017/S1463423618000439 [published Online First: 2018/07/02]
23. Sherratt FC, Marcus MW, Robinson J, et al. Electronic cigarette use and risk perception in a Stop Smoking Service in England. *Addict Res Theory* 2015;23(4):336-42. doi: 10.3109/16066359.2015.1006629
24. Brose LS, Brown J, Hitchman SC, et al. Perceived relative harm of electronic cigarettes over time and impact on subsequent use. A survey with 1-year and 2-year follow-ups. *Drug Alcohol Depend* 2015;157:106-11. doi: 10.1016/j.drugalcdep.2015.10.014
25. Mello S, Bigman CA, Sanders-Jackson A, et al. Perceived Harm of Secondhand Electronic Cigarette Vapors and Policy Support to Restrict Public Vaping: Results From a National Survey of US Adults. *Nicotine Tob Res* 2016;18(5):686-93. doi: 10.1093/ntr/ntv232
26. Government. S. The Scottish Health Survey: 2016 edition https://www.gov.scot/Resource/0052/00525472.pdf (accessed September 2018), 2016.

For peer review only

BMJ Open

# Views of prison staff in Scotland on the potential benefits and risks of e-cigarettes in smoke-free prisons: a qualitative focus group study


| | |
|---|---|
| Journal: | *BMJ Open* |
| Manuscript ID | bmjopen-2018-027799.R1 |
| Article Type: | Research |
| Date Submitted by the Author: | 18-Mar-2019 |
| Complete List of Authors: | Brown, Ashley; University of Stirling Institute for Social Marketing,<br>Sweeting , Helen ; University of Glasgow, MRC/CSO Social & Public Health Sciences Unit<br>Semple, Sean; University of Stirling, Institute of Social Marketing<br>Bauld, Linda; University of Edinburgh, Usher Institute of Population Health Sciences and Informatics<br>Demou, Evangelia; University of Glasgow, MRC/CSO Social & Public Health Sciences Unit<br>Logan, Greig; University of Glasgow, 4. Institute of Health and Wellbeing<br>Hunt, Kate; University of Stirling, Institute for Social Marketing |
| <b>Primary Subject Heading</b>: | Smoking and tobacco |
| Secondary Subject Heading: | Public health |
| Keywords: | PUBLIC HEALTH, Health policy < HEALTH SERVICES ADMINISTRATION & MANAGEMENT, QUALITATIVE RESEARCH |
| | |


SCHOLARONE™
Manuscripts

**Views of prison staff in Scotland on the potential benefits and risks of e-cigarettes in smoke-free prisons: a qualitative focus group study**

Ashley Brown, MA1

Helen Sweeting, PhD2

Sean Semple, PhD1

Linda Bauld, PhD3

Evangelia Demou, PhD2

Greig Logan, PhD4

Kate Hunt, PhD1,4

Corresponding author: Ashley Brown, a.l.brown@stir.ac.uk

(Text currently ~5240 without title page, abstract, strengths and limitations and declarations)

1. Institute for Social Marketing, Faculty of Health Sciences and Sport, University of Stirling, Pathfoot Building, Stirling, FK9 4LA.
2. MRC/CSO Social and Public Health Sciences Unit, 200 Renfield St, University of Glasgow, G2 3AX
3. Usher Institute, College of Medicine and Veterinary Medicine, University of Edinburgh.
4. Institute of Health and Wellbeing, University of Glasgow, Glasgow G12 8RZ



For peer review only
For peer review only - http://bmjopen.bmj.com/site/about/guidelines.xhtml

# ABSTRACT

## Objective

Electronic cigarettes (e-cigarettes) were introduced into all Scottish prisons in February 2018, some months after prisons began preparing for a smoking ban in November 2018. In 2016/2017, prison staff views on the potential benefits and risks of e-cigarettes were explored in advance of the introduction of: (1) a smoking ban and (2) e-cigarettes.

## Setting

Fourteen prisons in Scotland.

## Participants

Seventeen focus groups and two paired interviews were conducted with 132 staff in 14 Scottish prisons 4-9 months before plans for a smoking ban were announced in July 2017. Both smoking and non-smoking staff were invited to participate.

## Results

Prison staff highlighted three potential risks of e-cigarettes in smoke-free prisons: staff health risks from e-cigarette vapour; prisoner health risks from vaping; and risks to both groups from e-cigarette misuse, defects or accidents. Conversely, potential benefits of e-cigarettes in smoke-free prisons centred on: reducing smoking-related health harms to staff and prisoners; helping prisoners to manage without tobacco; and supporting staff to maintain safety and discipline in prison. Staff who participated in focus groups had limited experience of vaping and expressed some uncertainty and misunderstandings about e-cigarettes.


For peer review only



For peer review only - http://bmjopen.bmj.com/site/about/guidelines.xhtml

## Conclusion

Our findings highlight that scientific uncertainty, misunderstanding about vaping, the complexity of prisons as workplaces and prison tobacco control policy all have implications for staff perceptions of the potential place of e-cigarettes in smoke-free prisons. To alleviate staff concerns, there is a need for reliable information on e-cigarettes. Staff may also require reassurances on whether products are 'tamper proof', and rules about vaping indoors.

## STRENGTHS AND LIMITATIONS OF THIS STUDY

- To our knowledge, this is the first study to examine staff views on the potential benefits and risks of introducing e-cigarettes for prisoners in a smoke-free prison system, using data collected *prior to* the announcement of a smoking ban and before there was widespread experience of e-cigarette use in prisons.
- This study provides valuable insight into the potential place of e-cigarettes in a complex workplace setting in which staff were, on the one hand, still exposed to second-hand smoke, whilst on the other increasingly aware that they could be responsible for the management and care of vulnerable and/or challenging individuals subject to enforced smoking abstinence.
- Our results are based on analysis of rich qualitative data collected from a relatively large sample of prison staff from fourteen Scottish prisons, with varied smoking histories and experience of working with diverse prisoner groups.
- It is important to acknowledge that: the sample was self-selecting, few members of staff had direct experience of vaping, and staff views may have developed since these qualitative data were collected.

For peer review only

# INTRODUCTION

There is a growing consensus that using e-cigarettes (vaping) is safer than smoking conventional cigarettes. A review of the scientific evidence on e-cigarettes, conducted by the National Academies of Sciences, Engineering and Medicine,[1] reported "*conclusive evidence that completely substituting e-cigarettes for combustible tobacco cigarettes reduces users' exposures to numerous toxins and carcinogens present in combustible tobacco cigarettes*." A similar view on e-cigarettes has been reached by UK health organisations, including, for example, the Royal College of Physicians,[2] Public Health England[3 4] and NHS Health Scotland.[5] There is however, greater disagreement within academic and health communities over issues such as the effectiveness of e-cigarettes as a smoking cessation aid[6] and the potential influence of e-cigarettes on smoking norms and uptake among adults and young people.[1 7]

The contested nature of e-cigarettes is perhaps unsurprising. The introduction of a new technology is inevitably accompanied by a period of scientific uncertainty and debate about how to regulate technology in the absence of conclusive evidence about its health effects. The 'precautionary principle' may be relevant in situations of uncertainty. The World Health Organisation's definition of the principle centres on the idea that "…*scientific uncertainty should not be used as a reason to postpone preventive measures*".[8] The precautionary principle requires complex judgements (even conjectures) about the likely balance of potential benefits and risks of introducing different types of preventive measures vs. not implementing these measures.[9] With respect to e-cigarettes, Fairchild and Bayer[10] suggest that beliefs among some experts that e-cigarettes are a potential threat to health and require strict regulation are *"shaped implicitly by a precautionary impulse"*. On the other hand, it has been suggested that experts who believe that e-cigarettes are part of the solution to the challenge of tobacco control might be influenced by the principle of harm reduction.[10] Harm



For peer review only - http://bmjopen.bmj.com/site/about/guidelines.xhtml

reduction strategies are informed by a belief that steps should be taken to minimise harm from tobacco and other drug use in circumstances in which abstinence is not achievable.[11]

In the UK, it is illegal to smoke in most enclosed workplaces and public spaces. As both residential settings and workplaces, prisons have historically adopted a distinctive approach to tobacco control, including partial exemption from smoke-free laws in the UK.[12] However, ongoing concerns about Second Hand Smoke (SHS) exposures are one factor in recent decisions by the UK and Scottish governments to extend smoke-free policies to all indoor and outdoor areas of prisons in Wales and England (rolling out from 2016) and Scotland (from 30th November 2018 in all 15 prisons).

E-cigarettes have been available for prisoners to buy in some English and Welsh prisons from 2016 and were first made available for prisoners to buy in all Scottish prisons in early 2018 (~10-15 months after the data presented in this paper were collected). The potential for e-cigarettes to help some prisoners to remain smoke-free is recognised by organisations such as NHS Health Scotland,[13] while other commentators have discussed the potential negative health and organisational effects of selling e-cigarettes in prisons which have implemented smoking bans.[14 15]

Commentary and research on e-cigarettes and their place in smoke-free environments has largely focused on the opinions of public health experts and the general public.[16 17] In-depth qualitative research examining employees' views on vaping in particular settings is required to help with the development of acceptable and effective workplace policies and measures on e-cigarettes. We believe the Tobacco In Prisons study (TIPs), as reported here, is one of the first studies to address specific evidence gaps in respect of e-cigarettes in one workplace, prisons, and to investigate views on the potential role of e-cigarettes in accompanying the removal of tobacco across a country's prison system. In this qualitative paper, we present



For peer review only - http://bmjopen.bmj.com/site/about/guidelines.xhtml

prison staff views, using data from Phase 1 of TIPs. These data were collected from 132 prison staff in 14 Scottish prisons several months before the July 2017 announcement that a comprehensive ban on smoking would be introduced from 30th November 2018 and prior to any significant policy debate in Scotland about the sale of e-cigarettes to prisoners. This paper extends previous reporting of prison staff and prisoner views on prison smoking bans, which only includes brief mention of the potential place of e-cigarettes in smoke-free prisons.[18] Here, we use the staff focus group data to explore in detail staff views on the specific benefits and risks of e-cigarettes. The research could help with the development of strategies in respect of e-cigarettes in prison and so support the successful introduction of smoke-free policies, and help reduce tobacco-related harms, not just in Scotland (where prisons have recently gone smoke-free) but in other jurisdictions that are considering implementing bans in the future.

# METHODS

Data were collected prior to the announcement of plans to implement a smoking ban in Scottish prisons. At the time of data collection, prisoners were allowed to smoke tobacco in cells and outdoor areas; staff could not smoke on prison premises. Nobody (staff, prisoners or visitors) was permitted to vape in Scottish prisons during the period in which these data were collected (November 2016-April 2017).

The study was approved by the SPS Research Access and Ethics Committee and University of Glasgow's College of Social Sciences Ethics Committee (ref number relating to staff data: 400150214).

## Patient and Public Involvement

The TIPs study was designed to ensure that the views of prisoners and prison staff, as expressed to a research team who were independent of the prison service, could be heard at



For peer review only - http://bmjopen.bmj.com/site/about/guidelines.xhtml

different stages of the process of moving towards smoke-free prisons in Scotland. At all stages of the study, a Research Advisory Group which included staff from various parts of the prison service and representation from unions representing prison staff, has given extensive feedback on the overall design of the study and on study materials (including topic guides for qualitative interviews/focus groups).

## Sampling and recruitment

As reported elsewhere,[18] 17 focus groups and two paired interviews (hereafter referred to collectively as 'focus groups') were conducted with staff from 14 Scottish prisons who had been recruited through a point of contact in each prison. The reason for carrying out paired interviews on two occasions was that other prison staff who were due to participate in the focus group were unable to attend at short notice. We asked the point of contact to invite around eight prison staff to participate in a focus group with other staff from the same prison. To enable the research to explore the diversity of views on smoking in prisons and prison smoking bans within and between establishments it was explained that ideally we would like the focus groups to include both smoking and non-smoking staff in a range of work roles. While we had limited control over how focus groups were assembled by the prison point of contact, most displayed some diversity with respect to staff smoking status. Across the sample of 132 staff, 78 had never smoked (NS); 30 were ex-smokers (ExS), and 11 currently used tobacco cigarettes (S). The smoking status for 13 participants is not known (NK). Eight staff reported having ever used an e-cigarette: five were currently vaping (V) and three were no longer vaping (ExV). Although it was not possible to record information on staff job roles consistently, the majority of those who took part were Scottish Prison Service staff, while some worked in the prison for other agencies, such as the National Health Service. Scottish Prison Service staff were a mix of residential, operational and instructor officers, managerial roles and administrative posts. The 14 prisons in which staff were working were varied with



For peer review only - http://bmjopen.bmj.com/site/about/guidelines.xhtml

respect to: prisoner population (e.g. sex, age, and sentence length), capacity, security status, and prison architecture.

Quotations are included to illustrate key perspectives, indicating the prison code, focus group and smoking status of each speaker (e.g. KA04 S=prison K, group A, participant 04, Smoker). Codes were randomly allocated to prisons by the research team specifically for this paper to protect anonymity.

## Data collection

Focus groups were chosen for this study as they are well suited to understanding the diversity of viewpoints on a subject and how opinions are shaped by varying personal, environmental and social factors. Focus groups (range 5-12 participants) and the two paired interviews were conducted between November 2016 and April 2017 by a member of the TIPs research team. They were carried out in a room in each prison chosen by the point of contact. The topic guide covered: smoking and exposures to SHS within prisons; smoking norms and prevalence within prison; the 'culture' of smoking within prisons; management of nicotine addiction (including e-cigarettes) in prisons and wider society; and opinions on rules on smoking. Specific areas for discussion on e-cigarettes included: whether staff had used e-cigarettes or knew others who used e-cigarettes; opinions about e-cigarettes in general; views on what might be good or bad about prisoners or staff vaping in prison; and opinions on any issues which might be raised by allowing vaping in prisons. The researchers formulated questions using their own words (often in response to issues raised by the groups), adjusted the order of topics as appropriate, prompted further discussion where relevant and invited staff to raise any points which they thought were pertinent.



For peer review only - http://bmjopen.bmj.com/site/about/guidelines.xhtml

### Data analysis and reporting

With written consent from participants, focus groups were audio recorded and transcribed verbatim. Transcripts were checked and de-identified prior to management of the data. TIPs researchers (KH, HS, ED and GL) who conducted the fieldwork developed a descriptive coding scheme to bring together data on similar topics in preparation for detailed analysis. This coding scheme was devised using a combination of inductive and deductive techniques. The task of coding transcripts was split between TIPs researchers. Due to the relatively large volume of qualitative data, summaries with digital links to the raw data for all content relating to e-cigarettes were subsequently produced by AB using the Framework function in NVivo software (QSR international). AB used the data summaries and raw data to conduct thematic analysis. The process involved identifying different dimensions of staff opinions on e-cigarettes, grouping together dimensions which were similar to create themes and sub-themes and naming the themes and sub-themes.[19] KH conducted independent analysis of the data, and other authors read a sample of the data to familiarise themselves. Emergent themes were discussed and revised until an interpretation was agreed on by all authors. This paper largely follows Standards for Reporting Qualitative Research (SRQR) guidelines (see online supplementary file 1).

## RESULTS

### Background: personal experience and expressed knowledge of e-cigarettes

Most staff who participated in the focus groups had little personal experience of e-cigarettes; only a small number (n=8/132) reported having ever tried vaping. Consequently, a recurring theme in the focus groups was staff reporting low levels of knowledge about e-cigarettes, contributing to a sense of uncertainty and confusion about vaping. One way in which this



For peer review only - http://bmjopen.bmj.com/site/about/guidelines.xhtml

emerged was in the different names which staff gave to e-cigarettes, including "*vaping machines*" (JA05 NS), "*vapour sticks*" (FA06 NS), and "*vape cigarette*" (HA03 ExS). Another way in which uncertainty about e-cigarettes emerged was in the frequent questions which staff asked each other or the interviewer during discussions:

HA05 NS *Isn't it quite expensive as well e-cigarettes, are they not quite expensive?*

HA02 NS *I don't know, I think they're getting cheaper, they were quite expensive.*

For peer review only

Staff also seemed to be unsure about how e-cigarettes work and whether there is more than one type of product. Staff occasionally muddled 'tobacco' and 'nicotine' when discussing e-cigarettes, although mistakes in terminology were generally corrected by another member of the group. There were also examples where staff appeared to confuse e-cigarettes with a nicotine inhaler.

Uncertainty was also expressed by some staff about what is known about the health risks and safety of e-cigarettes:

FA01 NS "*[w]ell I was just gonna ask, why are they banning vaping [in other contexts outside of prisons], is there something wrong with vaping? Because they're banning it in lots of different places…for some reason*"

In other cases, staff expressed greater awareness that there are gaps in the evidence on e-cigarettes, which was another source of uncertainty. It is interesting to note however, that some staff seemed unfamiliar with the pace at which knowledge has accumulated in recent years:

BA10 NS *The last time they reviewed the policy, there was still no reliable research on the health implications of e-cigarettes.*



For peer review only - http://bmjopen.bmj.com/site/about/guidelines.xhtml

Apparent misunderstandings about e-cigarettes were also identified. These included a staff member believing that it might be more dangerous for non-smokers to take up e-cigarettes compared to conventional smoking and statements made as ‘facts’, such as that e-cigarettes “*emit ten carcinogens as opposed to 100 from ordinary cigarettes”* (CD27 ExS).

Hence, it was against this background that staff were evaluating the potential benefits and risks of vaping in prison, should tobacco be removed from the prisons at some future date. Overall, opinions around allowing e-cigarettes in smoke-free prisons included views which could be described as positive, and others which were highly negative, with some staff reporting that they did not feel sufficiently well informed to have an opinion of any sort. Although only eight participants had experience of vaping, those with direct experience of e-cigarettes generally acknowledged their potential benefits in smoke-free prisons. However, there were exceptions; for instance, less positive views were expressed by a staff member who had themselves stopped vaping due to concerns about potential adverse health effects of e-cigarette vapour.

The range of potential risks and benefits of e-cigarettes in smoke-free prisons as discussed by all participants in the focus groups are explored in detail below.

## Perceptions of potential risks of e-cigarettes in a smoke-free prison

Potential risks of e-cigarettes in a smoke-free prison centred on three sub-themes: potential risks to staff health from second-hand vapour; potential risks to prisoner health from vaping; and potential risks to staff and prisoners from device misuse, product defects or accidents.

### Potential risks to staff health from second-hand vapour



For peer review only - http://bmjopen.bmj.com/site/about/guidelines.xhtml

Both non-smoking and smoking staff could worry that they might be harmed by breathing the vapour from prisoners' e-cigarettes when they came to work. Several raised concerns that health risks from second-hand vapour exposures might be uncovered in the future:

*LA8 ExS* *'…not everybody will buy the good quality e-cigarettes so…what's in that [e-cigarette vapour]? You obviously don't know, so I think in years to come they'll [bystanders] end up having problems. Might not be as bad as cigarette smoke, but I think the time to come…when they start doing research like that.*

Consequently, there were some staff, who expressed beliefs that e-cigarettes should be prohibited in Scottish prisons as a protective measure. Precautionary thinking about second-hand vapour (and the use of e-cigarettes) appeared to be influenced by several factors. As discussed, some staff appeared aware that there are gaps in scientific understanding of the health effects of e-cigarettes and second-hand vapour.

*CC19 NS* *I don't think there's enough research been done on the e-cigarettes to see if you can get passive smoking through that either so I wouldn't be happy with smokers …*
*CC22 NS* *So ban it until you've proved it's safe.*
*INTERVIEWER* *So they're basically too new at the moment?*

*CC22 NS* *Yes, that's it. You don't allow it until you can prove it's safe. This is what has not happened with tobacco.*

Existing restrictions on vaping in many public places outside of the prison context appeared to reinforce perceptions that second-hand vapour might pose a danger to health:

*NA06, S/ExV* *It's the vapour itself. Which is why obviously it's been banned on trains and things like that, because they don't know enough about the vapour and the effect it might have on those around them. So you might be trading second hand smoke for second hand vapour. And it's…could be having exactly the same effect on people's lungs and everything else as the second hand smoke does, so…*



For peer review only - http://bmjopen.bmj.com/site/about/guidelines.xhtml

A desire to avoid past mistakes in relation to countries permitting the sale of cigarettes before the long-term health effects of smoking were known, perceptions that not enough is known about what is inside e-cigarettes and vapour, and beliefs that prisoners might use poor quality products are examples of further justifications given by staff for potentially prohibiting vaping in prisons.

## Potential risks to prisoner health from vaping

Some staff worried about potential risks to prisoner health from the use of e-cigarettes, given that the long-term effects of vaping are not yet fully known. For example, one member of staff stated, "*I just don't think we should be giving them [prisoners] something [e-cigarettes] as a substitute that we don't even know a hundred per cent about.*" (AA06 NS). Two other group members continued this discussion, saying:

*AA07 ExS* *Ten years down the line, we could all be smoking these vapes, and then we find out there's a risk.*

*AA01 NS* *A brain tumour, or something.*

Concerns about risks to prisoner health from vaping might also have been influenced by perceptions that e-cigarettes were being used, in wider society, as a long-term replacement for tobacco, rather than as a means to quit nicotine. There were some suggestions that prisoners might struggle to give up vaping and that 'addiction' to e-cigarettes was undesirable on health grounds. There was also a suggestion that it might be beneficial if prisoners had access to e-cigarettes on an interim (rather than permanent) basis to help them to manage cravings without forming a vaping habit. However, there were other staff who suggested that



For peer review only - http://bmjopen.bmj.com/site/about/guidelines.xhtml

this might be unfair for prisoners who do not want to become abstinent and challenging for staff to manage.

*B01, unknown* *'…because again comparing it with the community, a lot of people do go for the e-cigarette, but like say they get addicted to it, and I just feel that we're trying to create a healthier lifestyle, and especially if they are confined, if they're allowed to smoke an e-cigarette, we're not helping them [prisoners], because they'll just keep doing it, and you'll find that they'll smoke more and more and more, so how's that helping them with a healthier lifestyle, whereas we're trying to get them totally off that.*

Potential risks to staff and prisoners from device misuse, product defects or accidents

As misuse of items was perceived to be an integral part of prison culture, there was a significant amount of discussion about potential ways in which prisoners might attempt to find alternative uses for e-cigarettes.

NA06 S/ExV *Nothing's a hundred per cent tamper-proof and we're never going to find anything that's a hundred per cent tamper-proof…*

Specific concerns focussed on rechargeable devices and associated chargers and potential risk of devices being used to conceal contraband, 'smoke' illegal drugs (such as psychoactive substances) and charge prohibited mobile phones, for instance.

EB12 NS *I mean, it's an alternative. And obviously, for your second hand smoke, it's beneficial. But what could they do with these cartridges, and that sort of stuff? I mean, you're talking about people who can make anything out of anything…Is this just more stuff you're introducing to the jail, which they could use to do whatever?*



For peer review only - http://bmjopen.bmj.com/site/about/guidelines.xhtml

For peer review only

There were also questions about the potential for devices to leak, explode or catch fire in prison. For example, one member of staff stated that e-cigarettes had received 'bad press' for '*blowing up in people's faces and people maybe have them in their pocket and it leaks or something and it burns"* (CB01 NS).

## Perceptions of potential benefits of e-cigarettes in a smoke-free prison

While risks were identified, so were potential benefits of allowing e-cigarettes in a smoke-free prison. This included reducing harm to staff and prisoner health from smoking and SHS; helping prisoners to manage without tobacco, and the potential role of e-cigarettes in staff strategies to maintain safety and discipline in prison, as described below.

### Reducing harm to staff and prisoner health from smoking and SHS

E-cigarettes were perceived by some staff to contain fewer harmful chemicals compared with conventional cigarettes and thus to be likely to pose fewer risks to the health of users:

JA02 NS *I think they [e-cigarettes] are better for…smokers that want to come off smoking and they change from cigarettes to them because there are less carcinogens in but there is still something in them [e-cigarettes]. It has to be to create the vapour to carry the nicotine there's something in there.*

Some staff also believed that second-hand vapour might be less dangerous and unpleasant for bystanders, with one staff member even saying the smell produced by some e-liquids was "*quite nice*" (FA02, NK). Consequently, there was a suggestion that e-cigarettes might be beneficial for the health of everyone working and living in prisons when weighed against the dangers of smoking and SHS:



For peer review only - http://bmjopen.bmj.com/site/about/guidelines.xhtml

| | |
|---|---|
| FA02 unknown | *They could get the vapour thing, I don't know a lot about it, but vaping is safer than smoking. Why not let them vapour when they're in prison.* |
| FA05 NS | *I'm not being funny...* |
| FA02 unknown | *Better for us as well.* |
| FA05 NS | *...I would rather somebody was vaping in the jail, than smoking some of the crap they smoke.* |
| FA02 unknown | *Yeah.* |
| FA03 NS | *And it's got to be better for the smoker* |

## Helping prisoners to manage without tobacco

Some suggested that e-cigarettes could play a role in helping prisoners (including individuals who intended to smoke on their release from prison) to manage without tobacco should smoking be banned in prisons in the future. There was some discussion about the extent to which allowing prisoners to vape in prison was consistent with current practice in respect of treatment of prisoners addicted to other substances, such as illegal drugs like heroin.

| | |
|---|---|
| IA02 V | *"I totally agree [about the introduction of e-cigarettes into prisons]. I think it's been borne out with other…with the way we've treated other addictions, mainly methadone, I think there does need to be a substitute, it's by all accounts…the research thus far says it's a far, far cleaner substitute…* |

Lack of availability of 'medical' e-cigarettes in the UK, and uncertainties about the health effects of vaping were highlighted as points of difference between e-cigarettes and other 'substitute' products. Additionally, some implied that existing nicotine replacement products could fulfil a similar role to e-cigarettes.

## The potential role of e-cigarettes in staff strategies to maintain safety and discipline in prison

For peer review only



For peer review only - http://bmjopen.bmj.com/site/about/guidelines.xhtml

There were some perceptions that, in the absence of tobacco, nicotine substitutes such as e-cigarettes and nicotine replacement therapy might help staff with the management and care of prisoners who were unable to smoke, especially new arrivals into custody:

GA04 NS *"I think if I was offering some…prisoners, you know, there's your cigarette, I'm taking them off you, there's a lollypop or there's an e-cigarette I think I'd rather give them an e-cigarette"*

However, some expressed the view that it might be unfair if prisoners were permitted to vape in the future, since staff are not allowed to vape at work.

CA14 NS *I just think, I don't know nothing about them either, but then if staff aren't allowed to bring them in.*

CA15 Ex *Why should prisoners be allowed?*

Nicotine substitutes, alongside other measures, were also believed to have the potential to reduce organisational problems (e.g. incidents of indiscipline, threats to staff safety and operational stability) associated with prison smoking bans. However, there was some discussion about whether and how substitutes for smoking might make the imposition of a smoking ban more achievable and help to diffuse challenging situations in prisons.

KA02 NS *Well, I think if it's [a smoking ban] managed properly and an alternative [to tobacco] is offered, you know, whether it's a certain e-cigarette or patches or something, then yes, it [a smoking ban] could work, but I think to have an outright ban with no alternative in place would just cause a hell of a problem.*

## DISCUSSION

To our knowledge this is the first study in any country to investigate staff views on the potential benefits and risks of introducing e-cigarettes for prisoners collected across an entire prison system before a decision to implement smoke-free policy. This evidence can assist with the development of acceptable and effective measures in respect of e-cigarettes in



For peer review only - http://bmjopen.bmj.com/site/about/guidelines.xhtml

prisons and, in turn, support successful implementation of smoking bans and reduce tobacco-related harms, not just in Scotland but in other jurisdictions that are considering introducing smoke-free policy in the future.

We found evidence of discord among prison staff in Scotland about the overall balance of potential benefits and risks of e-cigarettes in a smoke-free prison; it is important to bear in mind that the data were collected before plans for a prison smoking ban were announced, and e-cigarettes were available for purchase. We found that concerns about as yet unknown potential risks to health from e-cigarettes and second-hand vapour led some staff to feel apprehensive about the prospect of prisoners vaping. It is understandable that some prison staff showed precautionary attitudes about the possibility of replacing one workplace hazard (SHS exposures) with another that they thought could potentially be hazardous (prisoner vaping). The likelihood that some prisoners would try to modify devices, thus possibly causing harm to themselves or others, was cited as another potential risk of e-cigarettes in prisons. Significant discussion among staff about potential e-cigarette misuse or accidents might be, at least partly, explained by prison officers' primary responsibilities for maintaining operational safety and concerns about use of psychoactive substances in UK prisons.[20]

By contrast, staff support for allowing e-cigarettes in a smoke-free prison could be interpreted with reference to the principle of harm reduction. While e-cigarettes were believed to carry some risk, these risks were thought by some staff to be smaller than the certain dangers of smoking and SHS and the potential adverse consequences of removing tobacco from prisons. Given ongoing challenges in respect of supporting individuals to abstain from drug and alcohol use in prison, it is understandable that some staff believed that a range of nicotine substitutes should be offered in a smoke-free prison to help in the management of smoking addiction.



For peer review only - http://bmjopen.bmj.com/site/about/guidelines.xhtml

The finding that some within the staff group had misgivings and questions about e-cigarettes in smoke-free prisons in 2016/2017 was also reflected in a TIPs online survey of prison staff conducted at a similar time. The staff survey showed that 74% of staff (strongly) agreed that 'prison smoking bans are a good idea'. The proportion who (strongly) agreed that 'prison smoking bans are ok if prisoners are allowed e-cigarettes or vapes' was 36%. The equivalent TIPs survey of prisoners, conducted in the same time period, found evidence of stronger support for e-cigarettes among prisoners: whilst only 22% of prisoners (strongly) agreed that 'prison smoking bans are a good idea', prisoners expressed greater acceptance of bans (48%) if e-cigarettes were made available.[18]

A key strength, and novel element, of this paper is that it is based on analysis of rich qualitative data collected from a relatively large sample of prison staff with diverse smoking histories and experiences of working in varied prison settings and with different groups of prisoners. We therefore believe that our results provide a good indication of staff perspectives on the key potential benefits and risks of e-cigarettes in a smoke-free prison. We were able to collect such comprehensive data from Scottish prisons through close partnership working with senior staff with a remit for health and wellbeing, and others (such as representatives of employee Unions), in the Scottish Prison Service, starting with discussion of research plans in the pre-grant period. In line with the study design,[21] the research helped to inform and verify implementation strategies for smoke-free prisons in Scotland by feeding back emergent findings from TIPs at monthly meetings of key stakeholders, including these findings on staff views on e-cigarettes, as prisons prepared to go smoke-free. We believe that the findings may be relevant to prisons in other countries who have adopted, or are considering, similar approaches to the UK on the regulation of tobacco and e-cigarettes.

Our study has four key limitations. First, focus group participants were self-selecting; it is notable that very few participating staff reported current/former vaping experience. A lower



For peer review only - http://bmjopen.bmj.com/site/about/guidelines.xhtml

rate of current use (~3%) of e-cigarettes among staff focus group participants compared with the general population in Scotland (7%)[22] is perhaps not surprising given that prison staff are not allowed to use e-cigarettes in their place of employment. Second, there are gaps in the information about participants, notably the number of years of experience of working in prisons, which could have provided additional useful context to the results. Third, in some focus groups there were individuals who expressed strong views about whether to allow e-cigarettes in prisons. While TIPs researchers tried to ensure that diverse and opposing positions were captured during the process of collecting and analysing data, it is possible that some people may not have wanted to express their views in front of colleagues. As such, some positions may not be fully reflected in our findings due to group dynamics.[23] Finally, it is important to acknowledge that staff viewpoints on e-cigarettes may have developed since these qualitative data were collected.

The findings of our research suggest a number of measures which, taken together, might increase staff awareness and understanding of e-cigarettes, and enhance support for their use in smoke-free prisons. This is important, since e-cigarettes might be beneficial for the transition to and ongoing management of smoke-free policy; in particular, they have the potential to enable prison staff to support strategies to increase prisoner motivation and capacity to achieve smoking abstinence, and ideally long-term cessation. By adding to the range of choices available to prisoners, e-cigarettes might help maximise the success, and health benefits, of smoke-free policy. Specifically, we suggest it would be beneficial if information on e-cigarettes were to be developed for prison settings which strikes a balance between taking a clear position on the relative harms of e-cigarettes compared to smoking tobacco or abstinence, while acknowledging limitations in the evidence. Such information might be valuable both for prison staff who are formally involved in health promotion work in prison (e.g. Physical Education Instructors), as well as for prison staff who might be



For peer review only - http://bmjopen.bmj.com/site/about/guidelines.xhtml

willing to provide opportunistic information and support in relation to e-cigarettes (and smoking behaviour) to prisoners. The findings support the sale of 'tamper proof' e-cigarettes in prison to protect staff and prisoner health. Additionally, they suggest it might be beneficial if frontline staff were offered training to enable them to swiftly identify when e-cigarettes are being used in ways which may cause serious injury to the user or other people, as well as opportunities to feedback to management about the implications of e-cigarettes for prison security. Rules on the indoor use of e-cigarettes in prison (as has happened in Scotland) might be prudent, given concerns among some staff about potential risks of exposure to e-cigarette vapour, and residual frustrations about the decision to partially exempt prisons from national smoke-free laws when they were introduced in 2006.

Future research conducted after e-cigarettes and smoke-free policies have been introduced in Scottish prisons is needed to increase understanding of the real world implications of: allowing prisoners to buy e-cigarettes in smoke-free prisons; the ongoing management of people who enter prison as smokers; prison security; smoking cessation provision; and staff and prisoner attitudes and health. Subsequent phases of TIPs and a complementary study will provide evidence in respect of these questions.

In conclusion, our findings highlight that gaps in scientific evidence on e-cigarettes, misunderstanding about vaping, the complexity of prisons as workplaces and the distinctive nature of prison tobacco control policy all have implications for staff perceptions of the risks and benefits of e-cigarettes in smoke-free prisons. Reliable information on e-cigarettes embedded in wider health promotion work in prison, sale of 'tamper proof' products and rules on vaping indoors might reduce staff concerns and so help in the successful implementation and long-term success of smoke-free prisons.

## CONTRIBUTIONSHIP STATEMENT



For peer review only - http://bmjopen.bmj.com/site/about/guidelines.xhtml

KH, HS, SS, ED and LB developed the study, with input from colleagues in the TIPs research team and the SPS Research Advisory Group. KH conducted most of the focus groups, with input from HS, ED and GL. AB and KH conducted independent initial analyses of the data, and AB conducted the more detailed framework analysis. AB drafted the manuscript and all revisions. All authors contributed to interpretation of the data and reviewing of the final paper.

## ACKNOWLEDGEMENTS

We are grateful to the staff who took part in the focus groups, and the Governors in Charge and staff at all 15 Scottish prisons who assisted with the study and facilitated access. We gratefully acknowledge the contribution of our co-investigators in TIPs research team to the overall design of the study (Dr Kathleen Boyd, Dr Philip Conaglen, Dr Peter Craig, Douglas Eadie, Professor Alastair Leyland, Professor Jill Pell,). We would particularly like to thank Sarah Corbett, Linda Dorward, Ruth Parker and members of the SPS Research Advisory Group for their helpful input during the design and conduct of this research, and to members of the SPS Smoke-free Stakeholder Advisory Group for their feedback on early findings of the study.

## COMPETING INTERESTS

None to declare

## FUNDING

The TIPs study was funded by the National Institute for Health Research Public Health Research Programme (project number 15/55/44). The views and opinions expressed are those of the authors and do not necessarily reflect those of the Public Health Programme, NIHR,



For peer review only - http://bmjopen.bmj.com/site/about/guidelines.xhtml

NHS or the Department of Health. HS and ED are also funded by UK Medical Research Council (MC_UU_12017/12, MC_PC_13027 to ED) and Chief Scientist Office (SPHSU-12)..

## DATA SHARING STATEMENT.

No additional data are available.

For peer review only

# REFERENCES

1. National Academies of Sciences EaM. Public Health Consequences of E-cigarettes: Conclusions by Outcome https://www.nap.edu/resource/24952/012318ecigaretteConclusionsbyOutcome.pdf (accessed August 2018), 2018.
2. Royal College of Physicians. Nicotine without Smoke: Tobacco harm reduction. https://www.rcplondon.ac.uk/projects/outputs/nicotine-without-smoke-tobacco-harm-reduction-0 (accessed June 2018), 2016.
3. Public Health England. E-cigarettes: a developing public health consensus Accessed online: https://www.gov.uk/government/publications/e-cigarettes-a-developing-public-health-consensus, 2016.
4. McNeill A, Brose LS, Calder R, et al. Evidence review of e-cigarettes and heated tobacco products 2018: A report commissioned by Public Health England. https://assets.publishing.service.gov.uk/government/uploads/system/uploads/attachment_data/file/684963/Evidence_review_of_e-cigarettes_and_heated_tobacco_products_2018.pdf (accessed September 2018): Public Health England 2018.
5. NHS Health Scotland. Consesus statement on e-cigarettes http://www.healthscotland.scot/media/1576/e-cigarettes-consensus-statement_sep-2017.pdf (accessed June 2018), 2017.
6. Villanti AC, Feirman SP, Niaura RS, et al. How do we determine the impact of e-cigarettes on cigarette smoking cessation or reduction? Review and recommendations for answering the research question with scientific rigor. *Addiction* 2018;113(3):391-404. doi: doi:10.1111/add.14020
7. Bauld L, MacKintosh A, Eastwood B, et al. Young People's Use of E-Cigarettes across the United Kingdom: Findings from Five Surveys 2015–2017. *Int J Environ Res Public Health* 2017;14(9):973.
8. Martuzzi M, Tickner JA. The precautionary principle: protecting public health, the environment and the future of our children http://www.euro.who.int/en/publications/abstracts/precautionary-principle-the-protecting-public-health,-the-environment-and-the-future-of-our-children (accessed June 2018): World Health Organisation 2004.
9. Public Health England. Use of e-cigarettes in public places and workplaces: Advice to inform evidence-based policy making. https://assets.publishing.service.gov.uk/government/uploads/system/uploads/attachment_data/file/534586/PHE-advice-on-use-of-e-cigarettes-in-public-places-and-workplaces.PDF (accessed June 2018), 2016.
10. Fairchild AL, Bayer R. Public health. Smoke and fire over e-cigarettes. *Science* 2015;347(6220):375-6. doi: 10.1126/science.1260761 [published Online First: 2015/01/24]
11. Lenton S, Single E. The definition of harm reduction. *Drug Alcohol Rev* 1998;17(2):213-20. doi: doi:10.1080/09595239800187011
12. Scottish Prison Service. Scotland's journey towards smoke-free prisons. http://www.sps.gov.uk/Corporate/Publications/Publication-4405.aspx (accessed September 2018), 2016.
13. NHS Health Scotland. Untitled https://www.scotphn.net/wp-content/uploads/2017/08/2016_10_06-Smokefree-prisons-and-e-cigs-Oct-2016.pdf (accessed June 2018), 2016.
14. Curry L, Lee YO, Rogers T. E-cigarettes made especially for inmates. *Tob Control* 2014;23(e2):e87-e88. doi: 10.1136/tobaccocontrol-2013-051535



For peer review only - http://bmjopen.bmj.com/site/about/guidelines.xhtml

15. Young-Wolff KC, Karan LD, Prochaska JJ. Electronic cigarettes in jails: A panacea or public health problem? *JAMA Psychiatry* 2015;72(2):103-04. doi: 10.1001/jamapsychiatry.2014.2224
16. Brose LS, McNeill A, Arnott D, et al. Restrictions on the use of e-cigarettes in public and private places-current practice and support among adults in Great Britain. *Eur J Public Health* 2017;27(4):729-36. doi: 10.1093/eurpub/ckw268
17. Majeed BA, Dube SR, Sterling K, et al. Opinions About Electronic Cigarette Use in Smoke-Free Areas Among U.S. Adults, 2012. *Nicotine Tob Res* 2015;17(6):675-81. doi: 10.1093/ntr/ntu235
18. Brown A, Sweeting H, Logan G, et al. Prison staff and prisoner views on a prison smoking ban: evidence from the Tobacco in Prisons Study. *Nicotine Tob Res* 2018 doi: 10.1093/ntr/nty092 [published Online First: 2018/05/17]
19. Spencer L, Ritchie J, O'Connor W, et al. Analysis in Practice In: Ritchie J, Lewis J, McNaughton Nicholls C, et al., eds. Qualitative Research Practice London: Sage 2014.
20. HM Chief Inspector of Prisons for England and Wales. Annual Report 2016–17. https://www.justiceinspectorates.gov.uk/hmiprisons/wp-content/uploads/sites/4/2017/07/HMIP-AR_2016-17_CONTENT_11-07-17-WEB.pdf (accessed August 2018), 2017.
21. Hunt K, Sweeting H, Semple S. Evaluating graduated progress towards, and impacts of, the implementation of indoor smokefree prison facilities in Scotland. The Tobacco in Prisons Study. Version 1.4 https://www.journalslibrary.nihr.ac.uk/programmes/phr/155544/#/. Accessed January 2018
22. Government. S. The Scottish Health Survey: 2016 edition https://www.gov.scot/Resource/0052/00525472.pdf (accessed September 2018), 2016.
23. Smithson J. Using and analysing focus groups: Limitations and possibilities. *International Journal of Social Research Methodology* 2000;3(2):103-19. doi: 10.1080/136455700405172



For peer review only - http://bmjopen.bmj.com/site/about/guidelines.xhtml

**Table 1: Standards for Reporting Qualitative Research (SRQR)**

| **Criteria** | |
|---|---|
| Title | P1 |
| Abstract | P2-3 |
| Problem formulation | P4-6 |
| Purpose or research questions | P2, 5-6 |
| Qualitative approach and research paradigm | P8 |
| Research characteristics and reflexivity | - |
| Context | P7-8 |
| Sampling strategy | P7-8 |
| Ethical issues pertaining to human subjects | P6 |
| Data collection methods | P8 |
| Data collection instruments and technologies | P8 |
| Units of study | P7-8 |
| Data processing | P9 |
| Data analysis | P9 |
| Techniques to enhance trustworthiness | P9 |
| Synthesis and interpretation | P9-17 |
| Links to empirical data | P9-17 |
| Integration with prior work, implications, transferability and contributions to the field | P17-21 |
| Limitations | P3, 19-20 |
| Conflicts of interest | P22 |
| Funding | P22 |

For peer review only

# Views of prison staff in Scotland on the potential benefits and risks of e-cigarettes in smoke-free prisons: a qualitative focus group study

| | |
|---|---|
| Journal: | *BMJ Open* |
| Manuscript ID | bmjopen-2018-027799.R2 |
| Article Type: | Research |
| Date Submitted by the Author: | 07-May-2019 |
| Complete List of Authors: | Brown, Ashley; University of Stirling Institute for Social Marketing, Sweeting , Helen ; University of Glasgow, MRC/CSO Social & Public Health Sciences Unit Semple, Sean; University of Stirling, Institute of Social Marketing Bauld, Linda; University of Edinburgh, Usher Institute of Population Health Sciences and Informatics Demou, Evangelia; University of Glasgow, MRC/CSO Social & Public Health Sciences Unit Logan, Greig; University of Glasgow, 4. Institute of Health and Wellbeing Hunt, Kate; University of Stirling, Institute for Social Marketing |
| <b>Primary Subject Heading</b>: | Smoking and tobacco |
| Secondary Subject Heading: | Public health |
| Keywords: | PUBLIC HEALTH, Health policy < HEALTH SERVICES ADMINISTRATION & MANAGEMENT, QUALITATIVE RESEARCH |
| | |

SCHOLARONE™
Manuscripts

# Views of prison staff in Scotland on the potential benefits and risks of e-cigarettes in smoke-free prisons: a qualitative focus group study

Ashley Brown, MA1

Helen Sweeting, PhD2

Sean Semple, PhD1

Linda Bauld, PhD3

Evangelia Demou, PhD2

Greig Logan, PhD4

Kate Hunt, PhD1,4

Corresponding author: Ashley Brown, a.l.brown@stir.ac.uk

(Text currently ~5240 without title page, abstract, strengths and limitations and declarations)

1. Institute for Social Marketing, Faculty of Health Sciences and Sport, University of Stirling, Pathfoot Building, Stirling, FK9 4LA.
2. MRC/CSO Social and Public Health Sciences Unit, 200 Renfield St, University of Glasgow, G2 3AX
3. Usher Institute, College of Medicine and Veterinary Medicine, University of Edinburgh.
4. Institute of Health and Wellbeing, University of Glasgow, Glasgow G12 8RZ



For peer review only - http://bmjopen.bmj.com/site/about/guidelines.xhtml

# ABSTRACT

## Objective

Electronic cigarettes (e-cigarettes) were introduced into all Scottish prisons in February 2018, some months after prisons began preparing in 2017 for a smoking ban implemented in November 2018. In 2016/2017, prison staff views on the potential benefits and risks of e-cigarettes were explored in advance of the introduction of: (1) a smoking ban and (2) e-cigarettes.

## Setting

Fourteen prisons in Scotland.

## Participants

Seventeen focus groups and two paired interviews were conducted with 132 staff in 14 Scottish prisons 4-9 months before plans for a smoking ban were announced in July 2017. Both smoking and non-smoking staff were invited to participate.

## Results

Prison staff highlighted three potential risks of e-cigarettes in smoke-free prisons: staff health risks from e-cigarette vapour; prisoner health risks from vaping; and risks to both groups from e-cigarette misuse, defects or accidents. Conversely, potential benefits of e-cigarettes in smoke-free prisons centred on: reducing smoking-related health harms to staff and prisoners; helping prisoners to manage without tobacco; and supporting staff to maintain safety and discipline in prison. Staff who participated in focus groups had limited experience of vaping and expressed some uncertainty and misunderstandings about e-cigarettes.


For peer review only

## Conclusion

Our findings highlight that scientific uncertainty, misunderstanding about vaping, the complexity of prisons as workplaces and prison tobacco control policy all have implications for staff perceptions of the potential place of e-cigarettes in smoke-free prisons. To alleviate staff concerns, there is a need for reliable information on e-cigarettes. Staff may also require reassurances on whether products are 'tamper proof', and rules about vaping indoors.

## STRENGTHS AND LIMITATIONS OF THIS STUDY

- To our knowledge, this is the first study to examine staff views on the potential benefits and risks of introducing e-cigarettes for prisoners in a smoke-free prison system, using data collected *prior to* the announcement of a smoking ban and before there was widespread experience of e-cigarette use in prisons.
- This study provides valuable insight into the potential place of e-cigarettes in a complex workplace setting in which staff were, on the one hand, still exposed to second-hand smoke, whilst on the other increasingly aware that they could be responsible for the management and care of vulnerable and/or challenging individuals subject to enforced smoking abstinence.
- Our results are based on analysis of rich qualitative data collected from a relatively large sample of prison staff from fourteen Scottish prisons, with varied smoking histories and experience of working with diverse prisoner groups.
- It is important to acknowledge that: the sample was self-selecting, few members of staff had direct experience of vaping, and staff views may have developed since these qualitative data were collected.



For peer review only - http://bmjopen.bmj.com/site/about/guidelines.xhtml

# INTRODUCTION

There is a growing consensus that using e-cigarettes (vaping) is safer than smoking conventional cigarettes. A review of the scientific evidence on e-cigarettes, conducted by the National Academies of Sciences, Engineering and Medicine,[1] reported "*conclusive evidence that completely substituting e-cigarettes for combustible tobacco cigarettes reduces users' exposures to numerous toxins and carcinogens present in combustible tobacco cigarettes*." A similar view on e-cigarettes has been reached by UK health organisations, including, for example, the Royal College of Physicians,[2] Public Health England[3 4] and NHS Health Scotland.[5] There is however, greater disagreement within academic and health communities over issues such as the effectiveness of e-cigarettes as a smoking cessation aid[6] and the potential influence of e-cigarettes on smoking norms and uptake among adults and young people.[1 7]

The contested nature of e-cigarettes is perhaps unsurprising. The introduction of a new technology is inevitably accompanied by a period of scientific uncertainty and debate about how to regulate technology in the absence of conclusive evidence about its health effects. The 'precautionary principle' may be relevant in situations of uncertainty. The World Health Organisation's definition of the principle centres on the idea that "…*scientific uncertainty should not be used as a reason to postpone preventive measures*".[8] The precautionary principle requires complex judgements (even conjectures) about the likely balance of potential benefits and risks of introducing different types of preventive measures vs. not implementing these measures.[9] With respect to e-cigarettes, Fairchild and Bayer[10] suggest that beliefs among some experts that e-cigarettes are a potential threat to health and require strict regulation are *"shaped implicitly by a precautionary impulse"*. On the other hand, it has been suggested that experts who believe that e-cigarettes are part of the solution to the



For peer review only - http://bmjopen.bmj.com/site/about/guidelines.xhtml

challenge of tobacco control might be influenced by the principle of harm reduction.[10] Harm reduction strategies are informed by a belief that steps should be taken to minimise harm from tobacco and other drug use in circumstances in which abstinence is not achievable.[11]

In the UK, it is illegal to smoke in most enclosed workplaces and public spaces. As both residential settings and workplaces, prisons have historically adopted a distinctive approach to tobacco control, including partial exemption from smoke-free laws in the UK.[12] However, ongoing concerns about Second Hand Smoke (SHS) exposures are one factor in recent decisions by the UK and Scottish governments to extend smoke-free policies to all indoor and outdoor areas of prisons in Wales and England[13] (rolling out from 2016) and Scotland (from 30th November 2018 in all 15 prisons).[14]

E-cigarettes have been available for prisoners to buy in some English and Welsh prisons from 2016 and were first made available for prisoners to buy in all Scottish prisons in early 2018 (~10-15 months after the data presented in this paper were collected). The potential for e-cigarettes to help some prisoners to remain smoke-free is recognised by organisations such as NHS Health Scotland,[15] while other commentators have discussed the potential negative health and organisational effects of selling e-cigarettes in prisons which have implemented smoking bans.[16 17]

Commentary and research on e-cigarettes and their place in smoke-free environments has largely focused on the opinions of public health experts and the general public.[18 19] In-depth qualitative research examining employees' views on vaping in particular settings is required to help with the development of acceptable and effective workplace policies and measures on e-cigarettes. We believe the Tobacco In Prisons study (TIPs), as reported here, is one of the first studies to address specific evidence gaps in respect of e-cigarettes in one workplace, prisons, and to investigate views on the potential role of e-cigarettes in accompanying the

For peer review only

removal of tobacco across a country's prison system. In this qualitative paper, we present prison staff views, using data from Phase 1 of TIPs. These data were collected from 132 prison staff in 14 Scottish prisons several months before the July 2017 announcement that a comprehensive ban on smoking would be introduced from 30$^{th}$ November 2018 and prior to any significant policy debate in Scotland about the sale of e-cigarettes to prisoners. This paper extends previous reporting of prison staff and prisoner views on prison smoking bans, which only includes brief mention of the potential place of e-cigarettes in smoke-free prisons.[20] Here, we use the staff focus group data to explore in detail staff views on the specific benefits and risks of e-cigarettes. The research could help with the development of strategies in respect of e-cigarettes in prison and so support the successful introduction of smoke-free policies, and help reduce tobacco-related harms, not just in Scotland (where prisons have recently gone smoke-free) but in other jurisdictions that are considering implementing bans in the future.

# METHODS

Data were collected prior to the announcement of plans to implement a smoking ban in Scottish prisons. At the time of data collection, prisoners were allowed to smoke tobacco in cells and outdoor areas; staff could not smoke on prison premises. Nobody (staff, prisoners or visitors) was permitted to vape in Scottish prisons during the period in which these data were collected (November 2016-April 2017).

The study was approved by the SPS Research Access and Ethics Committee and University of Glasgow's College of Social Sciences Ethics Committee (ref number relating to staff data: 400150214).

## Patient and Public Involvement



For peer review only - http://bmjopen.bmj.com/site/about/guidelines.xhtml

The TIPs study was designed to ensure that the views of prisoners and prison staff, as expressed to a research team who were independent of the prison service, could be heard at different stages of the process of moving towards smoke-free prisons in Scotland. At all stages of the study, a Research Advisory Group which included staff from various parts of the prison service and representation from unions representing prison staff, has given extensive feedback on the overall design of the study and on study materials (including topic guides for qualitative interviews/focus groups).

## Sampling and recruitment

As reported elsewhere,[20] 17 focus groups and two paired interviews (hereafter referred to collectively as ‘focus groups’) were conducted with staff from 14 Scottish prisons who had been recruited through a point of contact in each prison. The reason for carrying out paired interviews on two occasions was that other prison staff who were due to participate in the focus group were unable to attend at short notice. We asked the point of contact to invite around eight prison staff to participate in a focus group with other staff from the same prison. To enable the research to explore the diversity of views on smoking in prisons and prison smoking bans within and between establishments it was explained that ideally we would like the focus groups to include both smoking and non-smoking staff in a range of work roles. While we had limited control over how focus groups were assembled by the prison point of contact, most displayed some diversity with respect to staff smoking status. Across the sample of 132 staff, 78 had never smoked (NS); 30 were ex-smokers (ExS), and 11 currently used tobacco cigarettes (S). The smoking status for 13 participants is not known (NK). Eight staff reported having ever used an e-cigarette: five were currently vaping (V) and three were no longer vaping (ExV). Although it was not possible to record information on staff job roles consistently, the majority of those who took part were Scottish Prison Service staff, while some worked in the prison for other agencies, such as the National Health Service. Scottish



For peer review only - http://bmjopen.bmj.com/site/about/guidelines.xhtml

Prison Service staff were a mix of residential, operational and instructor officers, managerial roles and administrative posts. The 14 prisons in which staff were working were varied with respect to: prisoner population (e.g. sex, age, and sentence length), capacity, security status, and prison architecture.

Quotations are included to illustrate key perspectives, indicating the prison code, focus group and smoking status of each speaker (e.g. KA04 S=prison K, group A, participant 04, Smoker). Codes were randomly allocated to prisons by the research team specifically for this paper to protect anonymity.

## Data collection

Focus groups were chosen for this study as they are well suited to understanding the diversity of viewpoints on a subject and how opinions are shaped by varying personal, environmental and social factors. Focus groups (range 5-12 participants) and the two paired interviews were conducted between November 2016 and April 2017 by a member of the TIPs research team. They were carried out in a room in each prison chosen by the point of contact. The topic guide covered: smoking and exposures to SHS within prisons; smoking norms and prevalence within prison; the 'culture' of smoking within prisons; management of nicotine addiction (including e-cigarettes) in prisons and wider society; and opinions on rules on smoking. Specific areas for discussion on e-cigarettes included: whether staff had used e-cigarettes or knew others who used e-cigarettes; opinions about e-cigarettes in general; views on what might be good or bad about prisoners or staff vaping in prison; and opinions on any issues which might be raised by allowing vaping in prisons. The researchers formulated questions using their own words (often in response to issues raised by the groups), adjusted the order of topics as appropriate, prompted further discussion where relevant and invited staff to raise any points which they thought were pertinent.

For peer review only

## Data analysis and reporting

With written consent from participants, focus groups were audio recorded and transcribed verbatim. Transcripts were checked and de-identified prior to management of the data. TIPs researchers (KH, HS, ED and GL) who conducted the fieldwork developed a descriptive coding scheme to bring together data on similar topics in preparation for detailed analysis. This coding scheme was devised using a combination of inductive and deductive techniques. The task of coding transcripts was split between TIPs researchers. Due to the relatively large volume of qualitative data, summaries with digital links to the raw data for all content relating to e-cigarettes were subsequently produced by AB using the Framework function in NVivo software (QSR international). AB used the data summaries and raw data to conduct thematic analysis. The process involved identifying different dimensions of staff opinions on e-cigarettes, grouping together dimensions which were similar to create themes and sub-themes and naming the themes and sub-themes.[21] KH conducted independent analysis of the data, and other authors read a sample of the data to familiarise themselves. Emergent themes were discussed and revised until an interpretation was agreed on by all authors. This paper largely follows Standards for Reporting Qualitative Research (SRQR) guidelines (see online supplementary file 1).

# RESULTS

## Background: personal experience and expressed knowledge of e-cigarettes

Most staff who participated in the focus groups had little personal experience of e-cigarettes; only a small number (n=8/132) reported having ever tried vaping. Consequently, a recurring



For peer review only - http://bmjopen.bmj.com/site/about/guidelines.xhtml

theme in the focus groups was staff reporting low levels of knowledge about e-cigarettes, contributing to a sense of uncertainty and confusion about vaping. One way in which this emerged was in the different names which staff gave to e-cigarettes, including "*vaping machines*" (JA05 NS), "*vapour sticks*" (FA06 NS), and "*vape cigarette*" (HA03 ExS). Another way in which uncertainty about e-cigarettes emerged was in the frequent questions which staff asked each other or the interviewer during discussions:

HA05 NS *Isn't it quite expensive as well e-cigarettes, are they not quite expensive?*

HA02 NS *I don't know, I think they're getting cheaper, they were quite expensive.*

Staff also seemed to be unsure about how e-cigarettes work and whether there is more than one type of product. Staff occasionally muddled 'tobacco' and 'nicotine' when discussing e-cigarettes, although mistakes in terminology were generally corrected by another member of the group. There were also examples where staff appeared to confuse e-cigarettes with a nicotine inhaler.

Uncertainty was also expressed by some staff about what is known about the health risks and safety of e-cigarettes:

FA01 NS "*[w]ell I was just gonna ask, why are they banning vaping [in other contexts outside of prisons], is there something wrong with vaping? Because they're banning it in lots of different places…for some reason*"

In other cases, staff expressed greater awareness that there are gaps in the evidence on e-cigarettes, which was another source of uncertainty. It is interesting to note however, that some staff seemed unfamiliar with the pace at which knowledge has accumulated in recent years:



For peer review only - http://bmjopen.bmj.com/site/about/guidelines.xhtml

BA10 NS *The last time they reviewed the policy, there was still no reliable research on the health implications of e-cigarettes.*

Apparent misunderstandings about e-cigarettes were also identified. These included a staff member believing that it might be more dangerous for non-smokers to take up e-cigarettes compared to conventional smoking and statements made as 'facts', such as that e-cigarettes "*emit ten carcinogens as opposed to 100 from ordinary cigarettes"* (CD27 ExS).

Hence, it was against this background that staff were evaluating the potential benefits and risks of vaping in prison, in the event that tobacco was removed from the prisons at some future date. Overall, opinions around allowing e-cigarettes in smoke-free prisons included views which could be described as positive, and others which were highly negative, with some staff reporting that they did not feel sufficiently well informed to have an opinion of any sort. Although only eight participants had experience of vaping, those with direct experience of e-cigarettes generally acknowledged their potential benefits in smoke-free prisons. However, there were exceptions; for instance, less positive views were expressed by a staff member who had themselves stopped vaping due to concerns about potential adverse health effects of e-cigarette vapour.

The range of potential risks and benefits of e-cigarettes in smoke-free prisons as discussed by all participants in the focus groups are explored in detail below.

## Perceptions of potential risks of e-cigarettes in a smoke-free prison

Potential risks of e-cigarettes in a smoke-free prison centred on three sub-themes: potential risks to staff health from second-hand vapour; potential risks to prisoner health from vaping; and potential risks to staff and prisoners from device misuse, product defects or accidents.

Potential risks to staff health from second-hand vapour



For peer review only - http://bmjopen.bmj.com/site/about/guidelines.xhtml

Some non-smoking and smoking staff worried that they might be harmed by breathing the vapour from prisoners' e-cigarettes when they came to work. Several raised concerns that health risks from second-hand vapour exposures might be uncovered in the future:

*LA8 ExS* *'…not everybody will buy the good quality e-cigarettes so…what's in that [e-cigarette vapour]? You obviously don't know, so I think in years to come they'll [bystanders] end up having problems. Might not be as bad as cigarette smoke, but I think the time to come…when they start doing research like that.*

Consequently, there were some staff, who expressed beliefs that e-cigarettes should be prohibited in Scottish prisons as a protective measure. Precautionary thinking about second-hand vapour (and the use of e-cigarettes) appeared to be influenced by several factors. As discussed, some staff appeared aware that there are gaps in scientific understanding of the health effects of e-cigarettes and second-hand vapour.

*CC19 NS* *I don't think there's enough research been done on the e-cigarettes to see if you can get passive smoking through that either so I wouldn't be happy with smokers …*
*CC22 NS* *So ban it until you've proved it's safe.*
*INTERVIEWER* *So they're basically too new at the moment?*

*CC22 NS* *Yes, that's it. You don't allow it until you can prove it's safe. This is what has not happened with tobacco.*

Existing restrictions on vaping in many public places outside of the prison context appeared to reinforce perceptions that second-hand vapour might pose a danger to health:

*NA06, S/ExV* *It's the vapour itself. Which is why obviously it's been banned on trains and things like that, because they don't know enough about the vapour and the effect it might have on those around them. So you might be trading second hand smoke for second hand vapour. And it's…could be having exactly the same effect on people's lungs and everything else as the second hand smoke does, so…*

A desire to avoid past mistakes in relation to countries permitting the sale of cigarettes before the long-term health effects of smoking were known, perceptions that not enough is known

For peer review only

about what is inside e-cigarettes and vapour, and beliefs that prisoners might use poor quality products are examples of further justifications given by staff for potentially prohibiting vaping in prisons.

## Potential risks to prisoner health from vaping

Some staff worried about potential risks to prisoner health from the use of e-cigarettes, given that the long-term effects of vaping are not yet fully known. For example, one member of staff stated, "*I just don't think we should be giving them [prisoners] something [e-cigarettes] as a substitute that we don't even know a hundred per cent about.*" (AA06 NS). Two other group members continued this discussion, saying:

*AA07 ExS* *Ten years down the line, we could all be smoking these vapes, and then we find out there's a risk.*

*AA01 NS* *A brain tumour, or something.*

Concerns about risks to prisoner health from vaping might also have been influenced by perceptions that e-cigarettes were being used, in wider society, as a long-term replacement for tobacco, rather than as a means to quit nicotine. For instance, one staff member (AA07, ExS) said: *'I know loads of people that use them (e-cigarettes), and they say they're great. And you get addicted to that as well.'* There were some suggestions that prisoners might struggle to give up vaping and that 'addiction' to e-cigarettes was undesirable. There was also a suggestion that it might be beneficial if prisoners had access to e-cigarettes on an interim (rather than permanent) basis to help them to manage cravings without forming a vaping



For peer review only - http://bmjopen.bmj.com/site/about/guidelines.xhtml

288 habit. However, there were other staff who suggested that this might be unfair for prisoners
289 who do not want to become abstinent and challenging for staff to manage.

*B01, unknown* *'…because again comparing it with the community, a lot of people do go for the e-cigarette, but like say they get addicted to it, and I just feel that we're trying to create a healthier lifestyle, and especially if they are confined, if they're allowed to smoke an e-cigarette, we're not helping them [prisoners], because they'll just keep doing it, and you'll find that they'll smoke more and more and more, so how's that helping them with a healthier lifestyle, whereas we're trying to get them totally off that.*

290

291 Potential risks to staff and prisoners from device misuse, product defects or accidents

292 As misuse of items was perceived to be an integral part of prison culture, there was a
293 significant amount of discussion about potential ways in which prisoners might attempt to
294 find alternative uses for e-cigarettes.

NA06 S/ExV *Nothing's a hundred per cent tamper-proof and we're never going to find anything that's a hundred per cent tamper-proof…*

295

296 Specific concerns focussed on rechargeable devices and associated chargers and potential risk
297 of devices being used to conceal contraband, 'smoke' illegal drugs (such as psychoactive
298 substances) and charge prohibited mobile phones, for instance.

EB12 NS *I mean, it's an alternative. And obviously, for your second hand smoke, it's beneficial. But what could they do with these cartridges, and that sort of stuff? I mean, you're talking about people who can make anything out of anything…Is this just more stuff you're introducing to the jail, which they could use to do whatever?*

299



For peer review only - http://bmjopen.bmj.com/site/about/guidelines.xhtml

There were also questions about the potential for devices to leak, explode or catch fire in prison. For example, one member of staff stated that e-cigarettes had received 'bad press' for '*blowing up in people's faces and people maybe have them in their pocket and it leaks or something and it burns"* (CB01 NS).

## Perceptions of potential benefits of e-cigarettes in a smoke-free prison

While risks were identified, so were potential benefits of allowing e-cigarettes in a smoke-free prison. This included reducing harm to staff and prisoner health from smoking and SHS; helping prisoners to manage without tobacco, and the potential role of e-cigarettes in maintaining safety and discipline in prison, as described below.

### Reducing harm to staff and prisoner health from smoking and SHS

E-cigarettes were perceived by some staff to contain fewer harmful chemicals compared with conventional cigarettes and thus to be likely to pose fewer risks to the health of users:

JA02 NS *I think they [e-cigarettes] are better for…smokers that want to come off smoking and they change from cigarettes to them because there are less carcinogens in but there is still something in them [e-cigarettes]. It has to be to create the vapour to carry the nicotine there's something in there.*

Some staff also believed that second-hand vapour might be less dangerous and unpleasant for bystanders, with one staff member even saying the smell produced by some e-liquids was "*quite nice*" (FA02, NK). Consequently, there was a suggestion that e-cigarettes might be beneficial for the health of everyone working and living in prisons when weighed against the dangers of smoking and SHS:



For peer review only - http://bmjopen.bmj.com/site/about/guidelines.xhtml

| | |
|---|---|
| FA02 unknown | *They could get the vapour thing, I don't know a lot about it, but vaping is safer than smoking. Why not let them vapour when they're in prison.* |
| FA05 NS | *I'm not being funny...* |
| FA02 unknown | *Better for us as well.* |
| FA05 NS | *...I would rather somebody was vaping in the jail, than smoking some of the crap they smoke.* |
| FA02 unknown | *Yeah.* |
| FA03 NS | *And it's got to be better for the smoker* |

## Helping prisoners to manage without tobacco

Some suggested that e-cigarettes could play a role in helping prisoners (including individuals who intended to smoke on their release from prison) to manage without tobacco should smoking be banned in prisons in the future. There was some discussion about the extent to which allowing prisoners to vape in prison was consistent with current practice in respect of treatment of prisoners addicted to other substances, such as illegal drugs like heroin.

| | |
|---|---|
| IA02 V | *"I totally agree [about the introduction of e-cigarettes into prisons]. I think it's been borne out with other…with the way we've treated other addictions, mainly methadone, I think there does need to be a substitute, it's by all accounts…the research thus far says it's a far, far cleaner substitute…* |

Lack of availability of 'medical' e-cigarettes in the UK, and uncertainties about the health effects of vaping were highlighted as points of difference between e-cigarettes and other 'substitute' products. Additionally, some implied that existing nicotine replacement products could fulfil a similar role to e-cigarettes.

## The potential role of e-cigarettes in maintaining safety and discipline in prison



For peer review only - http://bmjopen.bmj.com/site/about/guidelines.xhtml

There were some perceptions that, in the absence of tobacco, nicotine substitutes such as e-cigarettes and nicotine replacement therapy might help staff with the management and care of prisoners who were unable to smoke, especially new arrivals into custody:

GA04 NS *"I think if I was offering some…prisoners, you know, there's your cigarette, I'm taking them off you, there's a lollypop or there's an e-cigarette I think I'd rather give them an e-cigarette"*

However, some expressed the view that it might be unfair if prisoners were permitted to vape in the future, since staff are not allowed to vape at work.

CA14 NS *I just think, I don't know nothing about them either, but then if staff aren't allowed to bring them in.*

CA15 Ex *Why should prisoners be allowed?*

Nicotine substitutes, alongside other measures, were also believed to have the potential to reduce organisational problems (e.g. incidents of indiscipline, threats to staff safety and operational stability) associated with prison smoking bans. However, there was some discussion about whether and how substitutes for smoking might make the imposition of a smoking ban more achievable and help to diffuse challenging situations in smoke-free prisons.

KA02 NS *Well, I think if it's [a smoking ban] managed properly and an alternative [to tobacco] is offered, you know, whether it's a certain e-cigarette or patches or something, then yes, it [a smoking ban] could work, but I think to have an outright ban with no alternative in place would just cause a hell of a problem.*

## DISCUSSION

To our knowledge this is the first study in any country to investigate staff views on the potential benefits and risks of introducing e-cigarettes for prisoners collected across an entire prison system before a decision to implement smoke-free policy. This evidence can assist



For peer review only - http://bmjopen.bmj.com/site/about/guidelines.xhtml

with the development of acceptable and effective measures in respect of e-cigarettes in prisons and, in turn, support successful implementation of smoking bans and reduce tobacco-related harms, not just in Scotland but in other jurisdictions that are considering introducing smoke-free policy in the future.

We found evidence of discord among prison staff in Scotland about the overall balance of potential benefits and risks of e-cigarettes in a smoke-free prison; it is important to bear in mind that the data were collected before plans for a prison smoking ban were announced, and before e-cigarettes were available for purchase. We found that concerns about as yet unknown potential risks to health from e-cigarettes and second-hand vapour led some staff to feel apprehensive about the prospect of prisoners vaping. It is understandable that some prison staff showed precautionary attitudes about the possibility of replacing one workplace hazard (SHS exposures) with another that they thought could potentially be hazardous (prisoner vaping). The likelihood that some prisoners would try to modify devices, thus possibly causing harm to themselves or others, was cited as another potential risk of e-cigarettes in prisons. Significant discussion among staff about potential e-cigarette misuse or accidents might be, at least partly, explained by prison officers' primary responsibilities for maintaining operational safety and concerns about use of psychoactive substances in UK prisons.[22]

By contrast, staff support for allowing e-cigarettes in a smoke-free prison could be interpreted with reference to the principle of harm reduction. While e-cigarettes were believed to carry some risk, these risks were thought by some staff to be smaller than the certain dangers of smoking and SHS and the potential adverse consequences of removing tobacco from prisons. Given ongoing challenges in respect of supporting individuals to abstain from drug and alcohol use in prison, it is understandable that some staff believed that a range of nicotine



For peer review only - http://bmjopen.bmj.com/site/about/guidelines.xhtml

substitutes should be offered in a smoke-free prison to help in the management of smoking addiction.

The finding that some within the staff group had misgivings and questions about e-cigarettes in smoke-free prisons in 2016/2017 was also reflected in a TIPs online survey of prison staff conducted at a similar time. The staff survey showed that 74% of staff (strongly) agreed that 'prison smoking bans are a good idea'. The proportion who (strongly) agreed that 'prison smoking bans are ok if prisoners are allowed e-cigarettes or vapes' was 36%. The equivalent TIPs survey of prisoners, conducted in the same time period, found evidence of stronger support for e-cigarettes among prisoners: whilst only 22% of prisoners (strongly) agreed that 'prison smoking bans are a good idea', prisoners expressed greater acceptance of bans (48%) if e-cigarettes were made available.[20]

A key strength, and novel element, of this paper is that it is based on analysis of rich qualitative data collected from a relatively large sample of prison staff with diverse smoking histories and experiences of working in varied prison settings and with different groups of prisoners. We therefore believe that our results provide a good indication of staff perspectives on the key potential benefits and risks of e-cigarettes in a smoke-free prison. We were able to collect such comprehensive data from Scottish prisons through close partnership working with senior staff with a remit for health and wellbeing, and others (such as representatives of employee Unions), in the Scottish Prison Service, starting with discussion of research plans in the pre-grant period. In line with the study design,[23] the research helped to inform and verify implementation strategies for smoke-free prisons in Scotland by feeding back emergent findings from TIPs at monthly meetings of key stakeholders, including these findings on staff views on e-cigarettes, as prisons prepared to go smoke-free. We believe that the findings may be relevant to prisons in other countries who have adopted, or are considering, similar approaches to the UK on the regulation of tobacco and e-cigarettes.



For peer review only - http://bmjopen.bmj.com/site/about/guidelines.xhtml

Our study has four key limitations. First, focus group participants were self-selecting; it is notable that very few participating staff reported current/former vaping experience. A lower rate of current use (~3%) of e-cigarettes among staff focus group participants compared with the general population in Scotland (7%)[24] is perhaps not surprising given that prison staff are not allowed to use e-cigarettes in their place of employment. Second, there are gaps in the information about participants, notably the number of years of experience of working in prisons, which could have provided additional useful context to the results. Third, in some focus groups there were individuals who expressed strong views about whether to allow e-cigarettes in prisons. While TIPs researchers tried to ensure that diverse and opposing positions were captured during the process of collecting and analysing data, it is possible that some people may not have wanted to express their views in front of colleagues. As such, some positions may not be fully reflected in our findings due to group dynamics.[25] Finally, it is important to acknowledge that staff viewpoints on e-cigarettes may have developed since these qualitative data were collected.

The findings of our research suggest a number of measures which, taken together, might increase staff awareness and understanding of e-cigarettes, and enhance support for their use in smoke-free prisons. This is important, since e-cigarettes might be beneficial for the transition to and ongoing management of smoke-free policy; in particular, they have the potential to enable prison staff to support strategies to increase prisoner motivation and capacity to achieve smoking abstinence, and ideally long-term cessation. By adding to the range of choices available to prisoners, e-cigarettes might help maximise the success, and health benefits, of smoke-free policy.  Specifically, we suggest it would be beneficial if information on e-cigarettes were to be developed for prison settings which strikes a balance between taking a clear position on the relative harms of e-cigarettes compared to smoking tobacco or abstinence, while acknowledging limitations in the evidence. Such information



For peer review only - http://bmjopen.bmj.com/site/about/guidelines.xhtml

might be valuable both for prison staff who are formally involved in health promotion work in prison (e.g. Physical Education Instructors), as well as for prison staff who might be willing to provide opportunistic information and support in relation to e-cigarettes (and smoking behaviour) to prisoners. The findings support the sale of 'tamper proof' e-cigarettes in prison to protect staff and prisoner health. Additionally, they suggest it might be beneficial if frontline staff were offered training to enable them to swiftly identify when e-cigarettes are being used in ways which may cause serious injury to the user or other people, as well as opportunities to feedback to management about the implications of e-cigarettes for prison security. Rules on the indoor use of e-cigarettes in prison (as has happened in Scotland) might be prudent, given concerns among some staff about potential risks of exposure to e-cigarette vapour, and possible residual frustrations about the decision to partially exempt prisons from national smoke-free laws when they were introduced in 2006.

Future research conducted after e-cigarettes and smoke-free policies have been introduced in Scottish prisons is needed to increase understanding of the real world implications of: allowing prisoners to buy e-cigarettes in smoke-free prisons; the ongoing management of people who enter prison as smokers; prison security; smoking cessation provision; and staff and prisoner attitudes and health. Subsequent phases of TIPs and a complementary study will provide evidence in respect of these questions.

In conclusion, our findings highlight that gaps in scientific evidence on e-cigarettes, misunderstanding about vaping, the complexity of prisons as workplaces and the distinctive nature of prison tobacco control policy all have implications for staff perceptions of the risks and benefits of e-cigarettes in smoke-free prisons. Reliable information on e-cigarettes embedded in wider health promotion work in prison, sale of 'tamper proof' products and rules on vaping indoors might reduce staff concerns and so help in the successful implementation and long-term success of smoke-free prisons.



For peer review only - http://bmjopen.bmj.com/site/about/guidelines.xhtml

## CONTRIBUTIONSHIP STATEMENT

KH, HS, SS, ED and LB developed the study, with input from colleagues in the TIPs research team and the SPS Research Advisory Group. KH conducted most of the focus groups, with input from HS, ED and GL. AB and KH conducted independent initial analyses of the data, and AB conducted the more detailed framework analysis. AB drafted the manuscript and all revisions. All authors contributed to interpretation of the data and reviewing of the final paper.

## ACKNOWLEDGEMENTS

We are grateful to the staff who took part in the focus groups, and the Governors in Charge and staff at all 15 Scottish prisons who assisted with the study and facilitated access. We gratefully acknowledge the contribution of our co-investigators in TIPs research team to the overall design of the study (Dr Kathleen Boyd, Dr Philip Conaglen, Dr Peter Craig, Douglas Eadie, Professor Alastair Leyland, Professor Jill Pell,). We would particularly like to thank Sarah Corbett, Linda Dorward, Ruth Parker and members of the SPS Research Advisory Group for their helpful input during the design and conduct of this research, and to members of the SPS Smoke-free Stakeholder Advisory Group for their feedback on early findings of the study.

## COMPETING INTERESTS

None to declare

## FUNDING

For peer review only



For peer review only - http://bmjopen.bmj.com/site/about/guidelines.xhtml

The TIPs study was funded by the National Institute for Health Research Public Health Research Programme (project number 15/55/44). The views and opinions expressed are those of the authors and do not necessarily reflect those of the Public Health Programme, NIHR, NHS or the Department of Health. HS and ED are also funded by UK Medical Research Council (MC_UU_12017/12, MC_PC_13027 to ED) and Chief Scientist Office (SPHSU-12).

## DATA SHARING STATEMENT.

No additional data are available.

For peer review only



For peer review only - http://bmjopen.bmj.com/site/about/guidelines.xhtml

# REFERENCES

1. National Academies of Sciences EaM. Public Health Consequences of E-cigarettes: Conclusions by Outcome https://www.nap.edu/resource/24952/012318ecigaretteConclusionsbyOutcome.pdf (accessed August 2018), 2018.
2. Royal College of Physicians. Nicotine without Smoke: Tobacco harm reduction. https://www.rcplondon.ac.uk/projects/outputs/nicotine-without-smoke-tobacco-harm-reduction-0 (accessed June 2018), 2016.
3. Public Health England. E-cigarettes: a developing public health consensus Accessed online: https://www.gov.uk/government/publications/e-cigarettes-a-developing-public-health-consensus, 2016.
4. McNeill A, Brose LS, Calder R, et al. Evidence review of e-cigarettes and heated tobacco products 2018: A report commissioned by Public Health England. https://assets.publishing.service.gov.uk/government/uploads/system/uploads/attachment_data/file/684963/Evidence_review_of_e-cigarettes_and_heated_tobacco_products_2018.pdf (accessed September 2018): Public Health England 2018.
5. NHS Health Scotland. Consesus statement on e-cigarettes http://www.healthscotland.scot/media/1576/e-cigarettes-consensus-statement_sep-2017.pdf (accessed June 2018), 2017.
6. Villanti AC, Feirman SP, Niaura RS, et al. How do we determine the impact of e-cigarettes on cigarette smoking cessation or reduction? Review and recommendations for answering the research question with scientific rigor. *Addiction* 2018;113(3):391-404. doi: doi:10.1111/add.14020
7. Bauld L, MacKintosh A, Eastwood B, et al. Young People's Use of E-Cigarettes across the United Kingdom: Findings from Five Surveys 2015–2017. *Int J Environ Res Public Health* 2017;14(9):973.
8. Martuzzi M, Tickner JA. The precautionary principle: protecting public health, the environment and the future of our children http://www.euro.who.int/en/publications/abstracts/precautionary-principle-the-protecting-public-health,-the-environment-and-the-future-of-our-children (accessed June 2018): World Health Organisation 2004.
9. Public Health England. Use of e-cigarettes in public places and workplaces: Advice to inform evidence-based policy making. https://assets.publishing.service.gov.uk/government/uploads/system/uploads/attachment_data/file/534586/PHE-advice-on-use-of-e-cigarettes-in-public-places-and-workplaces.PDF (accessed June 2018), 2016.
10. Fairchild AL, Bayer R. Public health. Smoke and fire over e-cigarettes. *Science* 2015;347(6220):375-6. doi: 10.1126/science.1260761 [published Online First: 2015/01/24]
11. Lenton S, Single E. The definition of harm reduction. *Drug Alcohol Rev* 1998;17(2):213-20. doi: doi:10.1080/09595239800187011
12. Scottish Prison Service. Scotland's journey towards smoke-free prisons. http://www.sps.gov.uk/Corporate/Publications/Publication-4405.aspx (accessed September 2018), 2016.
13. Selous A. Smoking in prisons https://www.gov.uk/government/speeches/smoking-in-prisons (accessed May 2019), 2015
14. Scottish Prison Service. Creating a Smoke Free Prison Environment http://www.sps.gov.uk/Corporate/News/Creating_a_Smoke_Free_Prison_Environment.aspx (accessed May 2018)2017



For peer review only - http://bmjopen.bmj.com/site/about/guidelines.xhtml

15. NHS Health Scotland. Untitled https://www.scotphn.net/wp-content/uploads/2017/08/2016_10_06-Smokefree-prisons-and-e-cigs-Oct-2016.pdf (accessed June 2018), 2016.
16. Curry L, Lee YO, Rogers T. E-cigarettes made especially for inmates. *Tob Control* 2014;23(e2):e87-e88. doi: 10.1136/tobaccocontrol-2013-051535
17. Young-Wolff KC, Karan LD, Prochaska JJ. Electronic cigarettes in jails: A panacea or public health problem? *JAMA Psychiatry* 2015;72(2):103-04. doi: 10.1001/jamapsychiatry.2014.2224
18. Brose LS, McNeill A, Arnott D, et al. Restrictions on the use of e-cigarettes in public and private places-current practice and support among adults in Great Britain. *Eur J Public Health* 2017;27(4):729-36. doi: 10.1093/eurpub/ckw268
19. Majeed BA, Dube SR, Sterling K, et al. Opinions About Electronic Cigarette Use in Smoke-Free Areas Among U.S. Adults, 2012. *Nicotine Tob Res* 2015;17(6):675-81. doi: 10.1093/ntr/ntu235
20. Brown A, Sweeting H, Logan G, et al. Prison staff and prisoner views on a prison smoking ban: evidence from the Tobacco in Prisons Study. *Nicotine Tob Res* 2018 doi: 10.1093/ntr/nty092 [published Online First: 2018/05/17]
21. Spencer L, Ritchie J, O'Connor W, et al. Analysis in Practice In: Ritchie J, Lewis J, McNaughton Nicholls C, et al., eds. Qualitative Research Practice London: Sage 2014.
22. HM Chief Inspector of Prisons for England and Wales. Annual Report 2016–17. https://www.justiceinspectorates.gov.uk/hmiprisons/wp-content/uploads/sites/4/2017/07/HMIP-AR_2016-17_CONTENT_11-07-17-WEB.pdf (accessed August 2018), 2017.
23. Hunt K, Sweeting H, Semple S. Evaluating graduated progress towards, and impacts of, the implementation of indoor smokefree prison facilities in Scotland. The Tobacco in Prisons Study. Version 1.4 https://www.journalslibrary.nihr.ac.uk/programmes/phr/155544/#/. Accessed January 2018

24. Government. S. The Scottish Health Survey: 2016 edition https://www.gov.scot/Resource/0052/00525472.pdf (accessed September 2018), 2016.
25. Smithson J. Using and analysing focus groups: Limitations and possibilities. *International Journal of Social Research Methodology* 2000;3(2):103-19. doi: 10.1080/136455700405172



For peer review only - http://bmjopen.bmj.com/site/about/guidelines.xhtml

**Table 1: Standards for Reporting Qualitative Research (SRQR)**

| | |
|---|---|
| **Title and abstract** | |
| **Title**- Concise description of the nature and topic of the study Identifying the study as qualitative or indicating the approach (e.g., ethnography, grounded theory) or data collection methods (e.g., interview, focus group) is recommended. | P1 |
| **Abstract** - Summary of key elements of the study using the abstract format of the intended publication; typically includes background, purpose, methods, results, and conclusions. | P2-3 |
| **Introduction** | |
| **Problem formulation** - Description and significance of the problem/phenomenon studied; review of relevant theory and empirical work; problem statement. | P4-6 |
| **Purpose or research questions** - Purpose of the study and specific objectives or questions. | P5-6 |
| **Methods** | |
| **Qualitative approach and research paradigm** - Qualitative approach (e.g., ethnography, grounded theory, case study, phenomenology, narrative research) and guiding theory if appropriate; identifying the research paradigm (e.g., postpositivist, constructivist/ interpretivist) is also recommended; rationale. | P8 |
| **Research characteristics and reflexivity** - Researchers' characteristics that may influence the research, including personal attributes, qualifications/experience, relationship with participants, assumptions, and/or presuppositions; potential or actual interaction between researchers' characteristics and the research questions, approach, methods, results, and/or transferability. | - |
| **Context** - Setting/site and salient contextual factors; rationale | P7-8 |
| **Sampling strategy** - How and why research participants, documents, or events were selected; criteria for deciding when no further sampling was necessary (e.g., sampling saturation); rationale. | P7-8 |
| **Ethical issues pertaining to human subjects** - Documentation of approval by an appropriate ethics review board and participant consent, or explanation for lack thereof; other confidentiality and data security issues. | P6 |
| **Data collection methods** - Types of data collected; details of data collection procedures including (as appropriate) start and stop dates of data collection and analysis, iterative process, triangulation of sources/methods, and modification of procedures in response to evolving study findings; rationale. | P8 |
| **Data collection instruments and technologies** - Description of instruments (e.g., interview guides, questionnaires) and devices (e.g., audio recorders) used for data collection; if/how the instrument(s) changed over the course of the study. | P8 |
| **Units of study** - Number and relevant characteristics of participants, documents, or events included in the study; level of participation (could be reported in results). | P7-8 |
| **Data processing** - Methods for processing data prior to and during analysis, including transcription, data entry, data management and security, verification of data integrity, data coding, and anonymization/de-identification of excerpts. | P9 |

For peer review only

| | |
|---|---|
| **Data analysis** - Process by which inferences, themes, etc., were identified and developed, including the researchers involved in data analysis; usually references a specific paradigm or approach; rationale. | P9 |
| **Techniques to enhance trustworthiness** - Techniques to enhance trustworthiness and credibility of data analysis (e.g., member checking, audit trail, triangulation); rationale. | P9 |
| **Results/findings** | |
| **Synthesis and interpretation** - Main findings (e.g., interpretations, inferences, and themes); might include development of a theory or model, or integration with prior research or theory. | P9-17 |
| **Links to empirical data** - Evidence (e.g., quotes, field notes, text excerpts, photographs) to substantiate analytic findings. | P9-17 |
| **Discussion** | |
| **Integration with prior work, implications, transferability and contributions to the field** - Short summary of main findings; explanation of how findings and conclusions connect to, support, elaborate on, or challenge conclusions of earlier scholarship; discussion of scope of application/generalizability; identification of unique contribution(s) to scholarship in a discipline or field. | P17-21 |
| **Limitations** - Trustworthiness and limitations of findings | P19-20 |
| **Conflicts of interest**- Potential sources of influence or perceived influence on study conduct and conclusions; how these were managed | P22 |
| **Funding** - Sources of funding and other support; role of funders in data collection, interpretation, and reporting. | P23 |

For peer review only
